# Characterization of the proneural gene regulatory network during mouse telencephalon development

**DOI:** 10.1186/1741-7007-6-15

**Published:** 2008-03-31

**Authors:** Julia M Gohlke, Olivier Armant, Frederick M Parham, Marjolein V Smith, Celine Zimmer, Diogo S Castro, Laurent Nguyen, Joel S Parker, Gerard Gradwohl, Christopher J Portier, François Guillemot

**Affiliations:** 1 Environmental Systems Biology Group, Laboratory of Molecular Toxicology, National Institute of Environmental Health Sciences, RTP, NC 27709, USA; 2 Division of Molecular Neurobiology, National Institute for Medical Research, The Ridgeway, Mill Hill, London, UK; 3 Constella Health Sciences, Durham, NC 27713, USA; 4 INSERM U682, Avenue Molière, 67200 Strasbourg, France

## Abstract

**Background:**

The proneural proteins Mash1 and Ngn2 are key cell autonomous regulators of neurogenesis in the mammalian central nervous system, yet little is known about the molecular pathways regulated by these transcription factors.

**Results:**

Here we identify the downstream effectors of proneural genes in the telencephalon using a genomic approach to analyze the transcriptome of mice that are either lacking or overexpressing proneural genes. Novel targets of Ngn2 and/or Mash1 were identified, such as members of the Notch and Wnt pathways, and proteins involved in adhesion and signal transduction. Next, we searched the non-coding sequence surrounding the predicted proneural downstream effector genes for evolutionarily conserved transcription factor binding sites associated with newly defined consensus binding sites for Ngn2 and Mash1. This allowed us to identify potential novel co-factors and co-regulators for proneural proteins, including Creb, Tcf/Lef, Pou-domain containing transcription factors, Sox9, and Mef2a. Finally, a gene regulatory network was delineated using a novel Bayesian-based algorithm that can incorporate information from diverse datasets.

**Conclusion:**

Together, these data shed light on the molecular pathways regulated by proneural genes and demonstrate that the integration of experimentation with bioinformatics can guide both hypothesis testing and hypothesis generation.

## Background

During development of the mammalian nervous system, neural progenitors within the neuroepithelium give rise sequentially to neuronal cells and glia. To achieve these well-orchestrated waves of differentiation, neuroepithelial progenitors are progressively constrained via specific extrinsic and intrinsic signals. By directly promoting the neuronal fate in neural progenitors, the proneural transcription factors of the bHLH family are essential regulators of neurogenesis from invertebrates to mammals [1]. Three proneural genes have been characterized to date in the embryonic mouse telencephalon: *Neurogenin1 *and *Neurogenin2 *are expressed in neuronal progenitors in dorsal telencephalon [2], which gives rise to the cerebral cortex, while *Mash1 *determines the fate of neuronal progenitors in the ventral telencephalon [3], giving rise to the basal ganglia. In addition to their role in the selection of neuronal progenitors within the neuroepithelium, vertebrate proneural genes have also been shown to specify neuronal subtype identities. Indeed, *Ngn2 *is necessary for the proper differentiation of excitatory glutamatergic projection neurons in the cerebral cortex, while *Mash1 *promotes the fate of GABA-ergic inhibitory interneurons in the basal ganglia [4]. Proneural transcription factors are thus critical regulators for both the initiation of neuronal differentiation and the specification of neurons into distinct regional subtypes.

In order to understand how *Ngn2 *and *Mash1 *regulate these two different aspects of neurogenesis we have developed a genomic approach incorporating the generation of microarray datasets of mice either lacking or overexpressing proneural genes, phylogenetic footprinting, and analysis using Bayesian statistical regression. The utility of Bayesian-based network analyses to determine predictive gene regulatory network structures has been demonstrated in *in vitro *and invertebrate models [5-7], but few attempts have been made using datasets derived from mammalian species due to the dependence of these techniques on a relatively large sample size [8]. However, this limitation may be eased through incorporation of data from other sources, such as prior information from the literature and sequence-based transcription factor binding site (TFBS) information [9].

Here, we initially identify a gene regulatory network (GRN) from a compilation of the current literature on proneural bHLH protein regulation of telencephalic development during neurogenesis. Next, microarray data from gain-of-function (GOF) analysis obtained after electroporation of either *Ngn2 *or *Mash1 *in the developing mouse dorsal or ventral telencephalon, respectively, were fused with previous and new microarray results from proneural loss-of-function (LOF) experiments [4]. This allowed us to identify novel targets of Ngn2 and Mash1, and to formally test literature-based GRN hypotheses through Bayesian statistical analysis of global gene expression patterns. A total of nine predicted proneural targets were confirmed by *in situ *RNA hybridization on brain slices from embryos lacking or overexpressing *Mash1 *or *Ngn2*, including several members of Notch signaling pathway (*Dll1*, *Hes5 *and *Mfng*) as well as the RNA binding protein *Elavl4 *(*HuC/D*). Members of the Wnt signaling pathway are predicted as targets of Ngn2 in the cerebral cortex. We then took advantage of the identification of putative downstream effectors of proneural genes to identify conserved binding sites for Mash1 and Ngn2, then scanned the sequence surrounding them for other conserved TFBSs. From this analysis, we predict Creb, Tcf/Lef, Pou-domain containing transcription factors, Sox9, and Mef2a as novel co-factors (binding at a short distance) and co-regulators (binding at further distance) of proneural proteins. Finally, a novel Bayesian-based algorithm was developed to compile the microarray data, the TFBS analysis, and the literature-based network, generating a proneural GRN for the developing mouse telencephalon.

## Results

### Literature-based network structure

**Figure 1:**
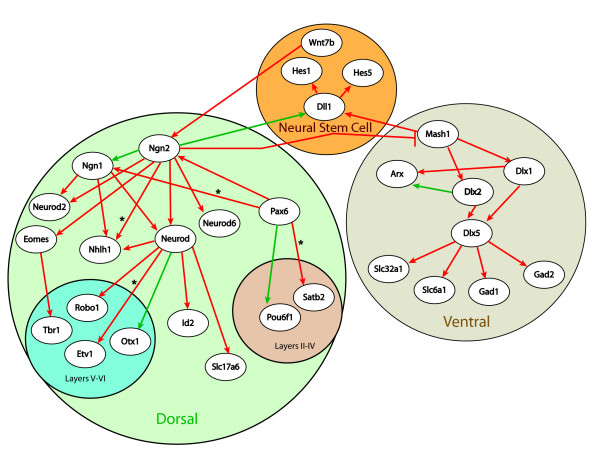
**Literature-based gene regulatory network describing proneural bHLH regulation of telencephalon neurogenesis**. Activations are identified with an arrow and repressions are identified with a barred line. Those connections that are significant based on the current microarray dataset are shown in red. Significant relationships were determined through analysis of the distribution of the strength of linkage parameter (β) after 500,000 MCMC simulations (Additional file 6). If more than 95% of the simulations have values above zero they are considered significant. A * denotes connections that were significant, but as inhibition.

A GRN that describes the current understanding of proneural bHLH interactions in the developing telencephalon was developed through a review of the literature (Figure 1). The reader is referred Additional file 1 and several important reviews[1,10,11] for more detailed descriptions of the experimental research underlying this literature-based GRN.

### GOF experiments

**Figure 2:**
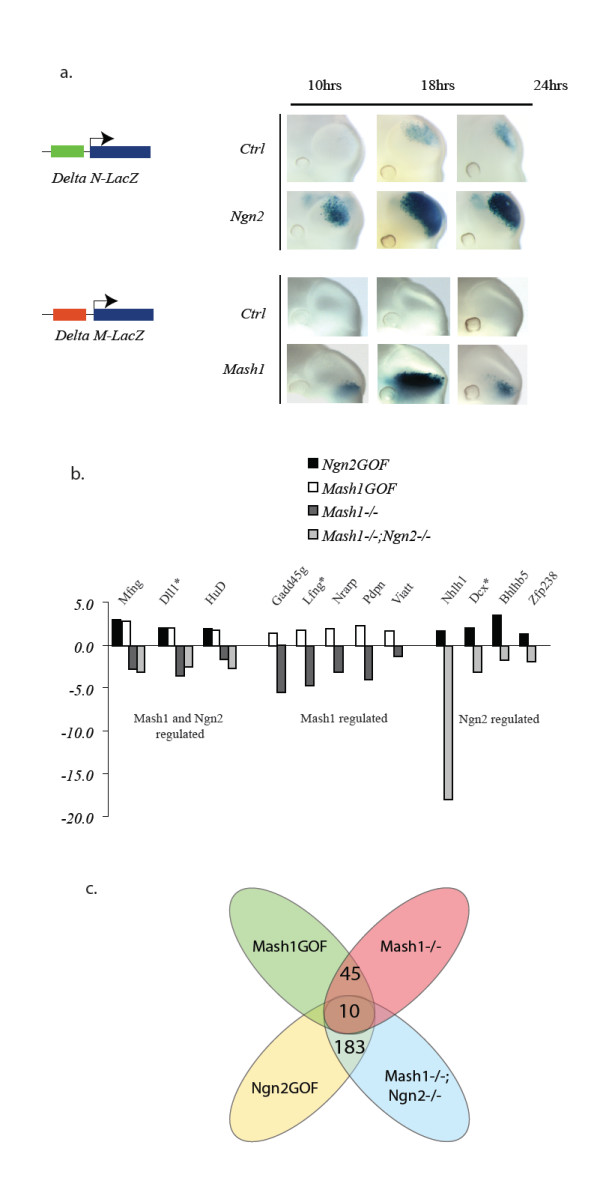
**Mash1 and Ngn2 GOF experiment**. (a) Mouse embryos at 10.5 days of development were electroporated in the telencephalic vesicle with *Mash1 *or *Ngn2 *expression vectors together with LacZ reporter constructs specific for either Ngn2 (0.27 kb DeltaN LacZ) or Mash1 (0.16 kb DeltaM LacZ) [12,79]. The two regulatory sequences used in these reporter transgenes are located within the 0.8 kb distal promoter region of the *Dll1 *gene [79]. Embryos were orientated during the electroporation to target the cortex (Ngn2 vector and 0.27 kb DeltaN LacZ reporter) or the basal ganglia (Mash1 vector and 0.16 kb DeltaM LacZ reporter). Ngn2 and Mash1 overexpression enhances the activity of the LacZ reporter, assessed by β-galactosidase staining, compared with endogenous proneural proteins (empty expressing vector: *Ctrl*). Efficiency of electroporation was assessed using a GFP expression vector and was similar in all electroporated embryos (not shown). (b) Fold changes of selected potential targets of Ngn2, Mash1, or both factors in *Ngn2 *GOF and *Mash1 *GOF experiments and in *Mash1 *mutant and *Mash1;Ngn2 *double mutant embryos, based on normalized microarray data. A * indicates known direct targets of *Mash1 *or *Ngn2*. (c) Putative targets of Mash1 and Ngn2 were identified through fusion of *Ngn2 *and *Mash1 *LOF and GOF microarray datasets. Common targets were identified as transcripts that were decreased in *Mash1 *LOF and *Mash1;Ngn2 *LOF experiments and increased in *Ngn2 *and *Mash1 *GOF experiments. Mash1 targets are those transcripts that were only decreased in *Mash1 *LOF and increased in *Mash1 *GOF. Ngn2 targets are those transcripts that were only decreased in *Ngn2/Mash1 *LOF and increased in *Ngn2 *GOF. A cut-off of 1.3-fold was used as described in more detail in Methods. A full list of predicted targets categorized by GO is available in Additional file 3.

To further identify genes regulated by the proneural factors Ngn2 and Mash1 in the embryonic telencephalon and complement existing LOF data [4], we developed a GOF approach using electroporation in a whole embryo culture. The feasibility of the GOF study was tested by injection of a *Ngn2*-expressing or *Mash1*-expressing vector in the telencephalic vesicles of E10.5 mouse embryos. The time course of *Ngn2 *and *Mash1 *overexpression was followed with *Dll1 *promoter-*lacZ *reporter transgenes monitoring Ngn2 and Mash1 activities, respectively (Figure 2(a); see also [12]). *LacZ *reporter activity was detected 10 h after electroporation of *Ngn2 *or *Mash1 *in the dorsal and ventral sides of telencephalic vesicles, respectively, and reached the highest level 18 h after proneural gene electroporation (Figure 2(a)). In contrast, activation by endogenous proneural proteins of the two reporters in embryos electroporated with an empty expression vector remained low. The efficiency of electroporation was similar in the control and *Ngn2*- and *Mash1*-electroporated cortices, as assessed by expression of a co-electroporated *GFP *plasmid before LacZ staining (Additional File 2 and data not shown). Large-scale electroporation experiments of *Ngn2 *in the dorsal telencephalon and *Mash1 *in the ventral telencephalon of E10.5 embryos were thus performed and, 18 h later, the electroporated tissue was processed for RNA probe preparation (see Methods).

### Identification of candidate Ngn2 and Mash1 target genes

We reasoned that by comparing the transcriptome of embryos overexpressing proneural genes with expression data obtained from current *Mash1 *LOF datasets and previously published *Ngn2 *LOF datasets [4], we would identify novel downstream effectors of Mash1 and/or Ngn2 in the developing telencephalon. For the study of Ngn2 targets, data from the cortex of *Mash1*-/-;*Ngn2*-/- double mutant mice was utilized rather than *Ngn2*-/- single mutants in order to avoid the compensation due to de-repression of *Mash1 *in the cortex of the later mutants [2]. Analysis of microarray data allowed us to split potential proneural targets into three groups (Figure 2(b) and 2(c)): (i) genes regulated by both Mash1 and Ngn2; (ii) genes regulated by Mash1 only; and (iii) genes regulated by Ngn2 only. A full list of predicted targets categorized by Gene Ontology (GO) is presented in Additional file 3.

Briefly, among the 10 common targets were 3 genes involved in Notch signaling (*Dll1*, *Hes5*, and *Mfng*) and the RNA binding protein *Elavl4 *expressed in maturing neurons [13]. Specific targets of Ngn2 include genes involved in signal transduction (42 genes), transcription factors (18 genes) such as the cortical differentiation factors *Nhlh1 *and *Bhlhb5 *[14], proteins with kinase/phosphatase activity (12 genes) such as the serine/threonine kinase *Dcx*, already shown to be directly regulated by Ngn2 in the cortex [15] and other cortical genes such as the cell adhesion molecule *Ephrin A4 *[16]. Interestingly, several components of the Wnt pathway are predicted targets of Ngn2 including *Wnt7b *[17], *DIX domain containing 1 *[18], *Fzd1, Fzd3*, and *Tax1bp3*. It is noteworthy that *NeuroD*, a gene considered as directly regulated by Ngn proteins [19], was decreased in the *Ngn2*-/- embryos, but was not significantly upregulated after *Ngn2 *electroporation in microarray experiments, suggesting that the window for analysis of electroporated embryos was not suitable. Indeed, *in situ *hybridization analysis showed that electroporation of *Ngn2 *does not induce *NeuroD *expression in the telencephalon at 24 h, but only at 48 h after electroporation (Additional file 2). A similar delay has been reported for induction of *NeuroD *by the related gene *Ngn3 *in the intestine [20]. This suggests either that *NeuroD *is not a direct target of Ngn2, or that its expression also requires another factor that is not present at the time of electroporation. Involvement of other genes in the regulation of *NeuroD *is supported by the lack of a dramatic change in *NeuroD *expression in *Ngn2 *null mutant telencephalon [21].

Finally, putative targets of Mash1 include transcription factors (seven genes) such as the LIM homeobox transcription factor *Isl1*, which is potentially involved in the specification of GABAergic projection neurons in the striatum [22], and factors involved in signal transduction such as the GABA vesicular transporter *Slc32a1/VIAAT *[23] and the gene *GP38/Podoplanin *[24], the Notch regulated protein *Nrarp *shown to destabilize the Notch intracellular domain (NICD) [25-27], and *Lfng*, a modulator of Notch signaling regulated directly by Mash1 in the telencephalon [12]. These putative target genes form the basis for subsequent bioinformatics and network analyses to predict novel co-factors, co-regulators, and GRN connectivity.

### Validation of predicted target genes

**Figure 3:**
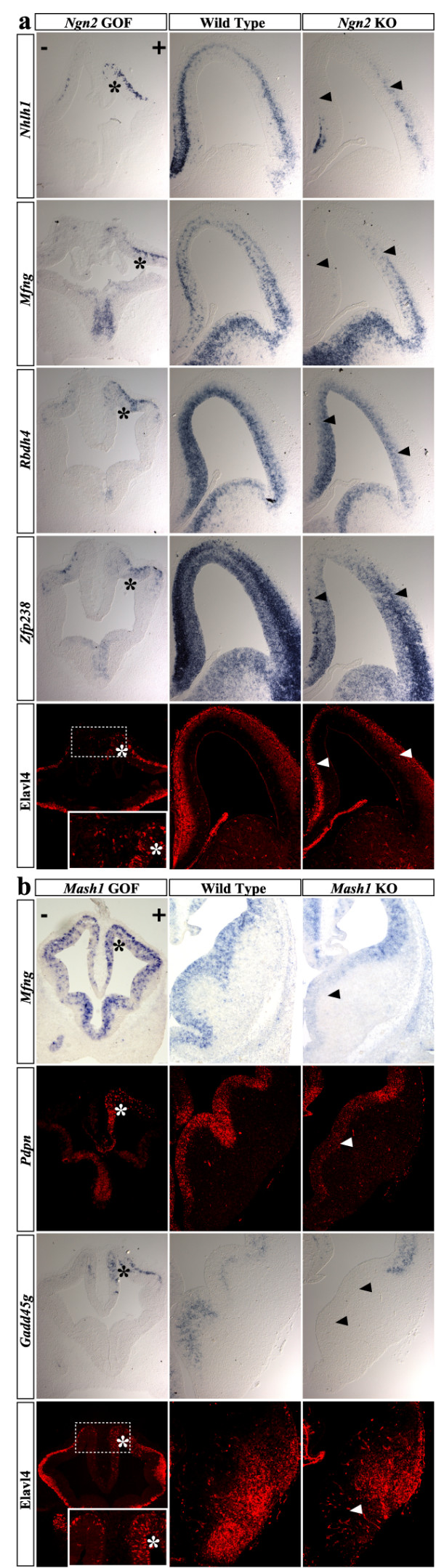
**Expression analysis of predicted Ngn2 and Mash1 targets through *in situ *hybridization of overexpressing and mutant embryos**. (a) Expression of the predicted Ngn2 targets *Nhlh1*, *zfp238*, *Rbdh4*, *Mfng*, and *Elavl4 *(the latter two are predicted common targets of Ngn2 and Mash1) in E10.5 embryos electroporated with a *Ngn2 *expression vector and cultivated for one day (left panels), in E13.5 wild-type embryos (middle panels), and in E13.5 *Ngn2 *mutant embryos (right panels). (b) Expression of the predicted Mash1 targets *Gadd45g*, *Pdpn*, *Mfng*, and *Elavl4 *in E10.5 embryos electroporated with a *Mash1 *expression vector and cultivated for one day (left panels), in E12.5 wild-type embryos (middle panels), and in E12.5 *Mash1 *mutant embryos (right panels). A * in the left panels indicates cells overexpressing the target genes. Arrowheads in the right panels indicate cells having downregulated the target genes.

To confirm the microarray data, several of the predicted novel targets of Ngn2 and Mash1 were validated through RNA *in situ *hybridization analysis of wild type and mutant embryos (*Ngn2-/- *and *Mash1-/-*), and of embryos overexpressing *Ngn2 *or *Mash1 *(Figure 3 and Additional file 4). The predicted *Ngn2 *targets *Nhlh1*, *Mfng*, and *Elavl4 *are mostly expressed at E13.5 in the mantle zone of the dorsal telencephalon, while *Rbdh4 *and *zfp238 *are also expressed in the ventricular zone. All five genes are downregulated in the medial part of the dorsal telencephalon of *Ngn2 *mutant embryos, indicating that *Ngn2 *is indeed required for normal expression of these genes (Figure 3(a)). We also show that *Ngn2 *can induce expression of the same predicted targets when overexpressed by electroporation in the telencephalon of E10.5 embryos. At this stage, their expression is barely detectable in neurons that begin to accumulate above the ventricular zone of the dorsal telencephalon (left telencephalic vesicles in Figure 3(a)). Expression of *Nhlh1*, *Mfng, zfp238*, *Rbdh4*, and *Elavl4 *is strongly upregulated after electroporation of a *Ngn2*-expressing vector in the dorsal telencephalon (right telencephalic vesicles in Figure 3(a), marked by *). Thus, both LOF and GOF analysis confirm that five predicted targets are indeed regulated by Ngn2 in the developing mouse telencephalon.

The predicted Mash1 targets *Mfng *and *Pdpn *are predominantly expressed in the ventricular zone of the ventral telencephalon at E12.5, while *Gadd45g *is predominantly expressed in the subventricular zone and *Elavl4 *in the mantle zone (*Mnfg *and *Elavl4 *are common candidate targets of *Mash1 *and *Ngn2*). All four genes are downregulated in the telencephalon of *Mash1 *mutant embryos particularly in the medial part of the ventral telencephalon, which is most severely affected in *Mash1 *mutant embryos [3] (Figure 3(b)). Ectopic expression of *Mash1 *in the dorsal telencephalon of E10.5 embryos strongly induces expression of the four genes (Figure 3(b)). Mash1 also rapidly induces expression of *Gadd45g *and *Mfng *in the embryonal carcinoma cell line P19, as detected by quantitative reverse transcription polymerase chain reaction (RT-PCR; see Additional file 5). These data suggest that many of the genes identified by microarray analysis of *Mash1 *and *Ngn2 *GOF and LOF experiments are indeed regulated by Mash1 and Ngn2.

### Bayesian analysis of literature-based GRN

Connectivity in the literature-based network structure was quantified based on the GOF and LOF microarray gene expression datasets. This Bayesian-based method removes the need to rely on fold change cut-offs by examining the strength of the predicted relationships based on concurrently evaluating variability in gene expression patterns of several genes of interest across control and perturbation experiments. Significant connections based on this analysis (the fifth percentile of the posterior density for β is greater than zero [8]) are highlighted in red in Figure 1. Our analysis predicts 86% (31/36) of the connections as significant based on the microarray dataset; however, four are considered inhibitory interactions instead of activations, suggesting possible feedback loops between *Pax6 *and *Ngn1*, between *Satb2*, *Ngn2 *and *Nhlh1*, as well as between *NeuroD *and *Etv1 *(Additional file 6). Specificity of our method was estimated through the application of 100 randomly generated datasets by permutation of gene labels using the entire microarray dataset. This analysis results in an estimated false positive rate of 8.2%, therefore the chances of seeing 31 significant connections out of 36 is very low (*p *< 0.001). Two of the connections originating from *Ngn2 *(to *Ngn1 *and *Dll1*) are not considered significant based on the current analysis. As the algorithm relies on linear relationships between genes, its inability to detect these two connections may be a result of the non-linear variability between *Ngn2 *and its targets that is created by the large increase in *Ngn2 *transcript levels after electroporation. In addition, the compensatory action of *Mash1 *in the dorsal telencephalon of the *Ngn2-/- *mutants further complicates the algorithm's ability to detect connectivity to common targets such as *Dll1*.

### Bioinformatics prediction of Ngn2 and Mash1 co-factors

The identification of potential downstream effectors of Mash1 and Ngn2 allowed us to examine the occurrence of defined TFBSs in the conserved sequence surrounding those genes. The E-box recognized by bHLH proteins is a degenerate 6 bp (base pair) motif (CANNTG). However, recent evidence suggests that Mash1 and Ngn2 have different preferences for the two central residues and that their consensus binding sequences extend even outside of the E-Box motif (see [12,28,29] and FG and DC, unpublished data). Based on these and other data, we defined different consensus binding sequences for Mash1 (GCAGSTGK or CAGSTG) and Ngn2 (CANTWG) (Additional file 7).

**Figure 4:**
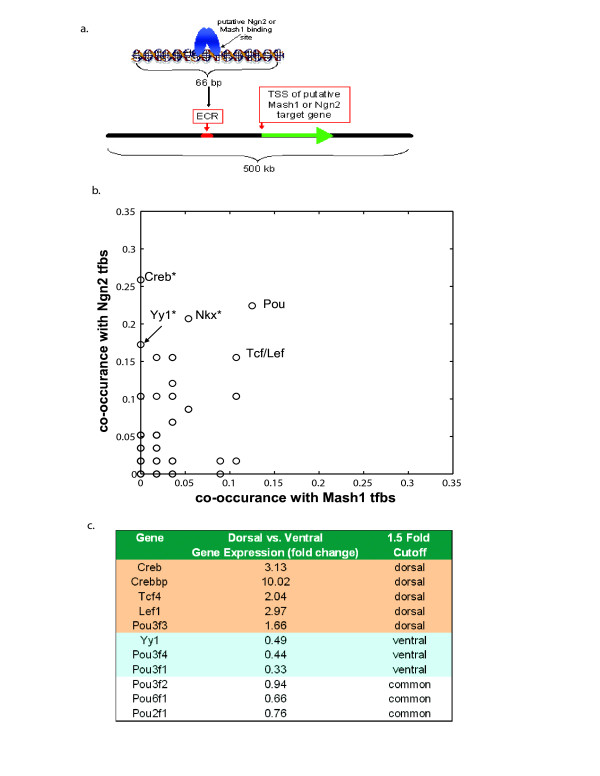
**Identification of potential co-factors for Ngn2 and Mash1 using bioinformatics approaches in comparative genomics**. (a) Putative Ngn2 and Mash1 bindings sites were identified in ECRs in a 500 kb region surrounding 34 Ngn2 and Mash1 target genes (list of target genes analyzed in Additional file 8). Other TFBSs were identified within 30 bp of the putative Ngn2 or Mash1 binding site. (b) TRANSFAC TFBSs that co-occurred with Ngn2 binding sites versus Mash1 binding sites surrounding Ngn2 target genes versus Mash 1 and common target genes, respectively. A total of 58 Ngn2 sites and 56 Mash1 binding sites were analyzed. A * denotes significantly enriched TFBSs in the sequence surrounding Ngn2 sites versus Mash1 sites (Fisher's exact two-sided test with *p *≤ 0.05). (c) Comparison of microarray gene expression values of potential Ngn2 and Mash1 co-factors in wild-type dorsal and ventral telencephalon tissues where column three presents results using a 1.5-fold change cut-off value for categorizing gene expression as either preferentially dorsal, ventral, or both (common).

To identify potential co-factors for Ngn2 or Mash1, we employed phylogenetic footprinting and TFBS search algorithms on a subset of the predicted targets based on the microarray dataset (Figure 4(a)). First, 58 conserved (human, mouse, chicken, frog, and fish) putative Ngn2 binding sites surrounding 11 of the predicted Ngn2 target genes and 56 conserved putative Mash1 binding sites surrounding 14 of the predicted Mash1 target genes and 6 of the common target genes were identified (see Methods for further details). Subsequently, we identified TFBS from the TRANSFAC library co-occurring specifically with the putative Ngn2 binding sites in Ngn2 targets or with the putative Mash1 binding sites in Mash1 and Mash1/Ngn2 common target genes using Fisher's exact two-sided test for significance (Figure 4(b)). No specific co-factors for Mash1 were identified in this analysis, whereas cAMP response element (CRE; bound by CRE binding protein (Creb)), Yy1, and Nkx binding sites were significantly enriched near Ngn2 binding sites when compared with the sequence around Mash1 binding sites (Figure 4(b)). Several of the predicted co-factors have dorsal/ventral restricted expression patterns in the embryonic telencephalon, consistent with a role in neuronal specification (Figure 4(c)).

### Bioinformatics prediction of Ngn2 and Mash1 co-regulators

Next we sought to predict potential co-regulators that may bind at regulatory modules independent from those that bind Ngn2 or Mash1. In particular, we were interested in identifying transcription factors that may confer regulation at modules closer to the TSS (transcription start site). In order to efficiently search for co-regulators, we identified evolutionary conserved TFBS (between human and mouse) using the CONFAC algorithm [30] to analyze a shorter sequence (10,000 bp) in the 5' flanking region of 31 predicted Mash1, Ngn2, and common targets (Additional file 8). To predict putative common co-regulators, we tested for enrichment of the identified TFBS in Ngn2 target genes and Mash1/common target genes compared with a set of 250 randomly selected genes using the Mann-Whitney statistical test. We identified 41 potential Ngn2- and Mash1-specific co-regulators by testing for TFBS enrichment in Ngn2 targets versus Mash1 targets and vice versa. We identified 14 putative co-regulators from these analyses after eliminating those transcription factors that are not expressed in either the dorsal or ventral developing telencephalon based on microarray transcript levels (Additional file 9). Predicted common co-regulators include E2f1, Tef, Nfy, Egr1, Hes1, and Pou-domain containing factors. Predicted co-regulators were specifically enriched in Ngn2 targets when compared with Mash1 and common targets include Sox9, Creb, Tcf4, Lef1, Mef2a, and Yy1. No transcription factors were significantly enriched when Mash1 target genes were compared against Ngn2 target genes. This approach identified all five of the predicted Ngn2 co-factors found in the previous analysis, as well as identifying several transcription factors that may co-regulate the putative Ngn2 and Mash1 targets at independent regulatory modules including E2f1, Egr1, Hes1, and Nfy in both Ngn2 and Mash1 targets and Sox9 and Mef2a in Ngn2 target genes.

Through interrogation of online databases of *in situ *hybridization in serial brain sections across development [31,32], we confirmed the expression of several predicted co-factors/co-regulators in the developing dorsal and ventral telencephalon (Additional file 10). Interestingly, several of the predicted co-factors/co-regulators, including Sox9, Crebbp, Creb1, Tcf4, Lef1, Pou6f1, Pou2f1, Pou3f1, Tef, Hes1, and E2f1, show appreciable expression in the ventricular zone of dorsal and/or ventral telencephalon, where proneural bHLH proteins are expressed. Furthermore, direct protein-protein interactions between proneural bHLH proteins and Crebbp, Tcf4, and Mef2a are reported in the human protein reference database [33].

### Bayesian network analysis with an informative prior structure

**Figure 5:**
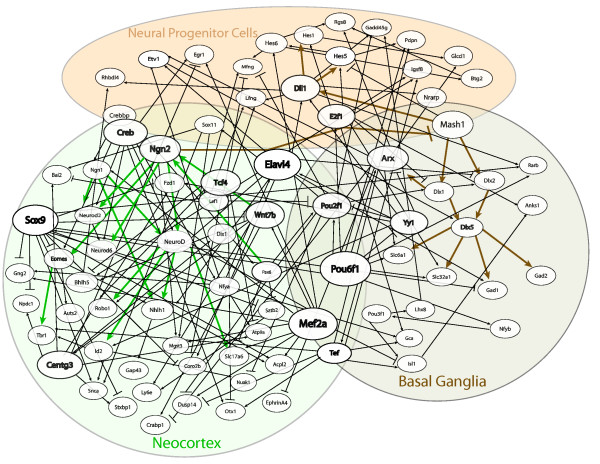
**Algorithm-based gene regulatory network structure for dorsal and ventral telencephalon development**. The Bayesian-based TAO-Gen algorithm was implemented with an informative prior structure to predict the optimal network structure based on the LOF and GOF microarray datasets, evolutionarily conserved TFBS data, prior literature-based knowledge, and spatial and time-specific expression patterns. To highlight the key regulators, the nodes representing genes predicted to be the parent of at least nine other genes are largest in size (Sox9, Mef2a, Elavl4 and Pou6f1), whereas those that are predicted to regulate at least five other genes are medium in size (Ngn2, Centg3, Tef, Tcf4, Wnt7b, Pou2f1, Yy1, Dll1, E2f1, Arx, and Creb). See Additional file 11 for matrix of connectivity and Methods for a more detailed description of algorithm.

To provide an integrated view of the network regulated by proneural genes, information obtained from the literature, novel expression data, and phylogenetic footprinting analyses were quantitatively linked through application of the Bayesian-based TAO-Gen algorithm [34] with the addition of an informative prior structure (Figure 5, Additional file 11). Nodes represented in the network include several candidate Mash1 and Ngn2 target genes from the fusion and sorting of the GOF/LOF microarray datasets, as well as candidate co-factors and co-regulators from the phylogenetic footprinting analyses. An informative prior structure considered all significant literature-based connections as required (diagrammed using thicker lines) and used the TFBS information from the phylogenetic footprinting analyses to weight connections in which TFBS information was found. For example, a conserved Sox9 TFBS was identified in the sequence surrounding *Lfng*, therefore a preference is given to Sox9 being a parent to *Lfng*, which results in a slightly higher probability that a linear relationship between these two genes will be significant in the posterior distribution. We have confirmed the reliability of the TAO-Gen algorithm as well as the utility of the informative prior structure through direct comparisons with results obtained from another Bayesian network algorithm [35,36], as well as with results obtained using TAO-Gen without an informative prior (Additional file 1).

The resulting Bayesian-based network structure predicts 174 linkages (27 of which are significant lit-based connections) between the set of 82 genes. Sox9, Mef2a, Elavl4, and Pou6f1 are predicted as the most prolific co-regulators of the target genes, with 14, 12, 9, and 12 children, respectively. Furthermore, our analysis predicts Creb1, Crebbp, and Yy1 as the most likely candidates for dorsally expressed Ngn2 co-factors, and supports a synergistic interaction between Pou-domain containing transcription factors and bHLH proneural proteins in the regulation of common target genes, which is consistent with previous studies [12]. We also identified several transcription factors as candidate co-regulators of Ngn2 target genes, including Hes1, Egr1, Nfy, Mef2a, Tef, and Sox9, whereas Pou6f1 is a predicted co-regulator of Mash1 target genes. Interestingly, these different transcription factors have been previously implicated in regulatory processes in other developmental contexts [37-42], consistent with a potential role in regulating neurogenesis during telencephalon development.

## Discussion

As the amount of experimental data grows and becomes more complex, the power of computational models to identify mechanisms of biological processes by integrating diverse sets of experimental data is being realized, particularly in organisms where high throughput perturbation analyses have been developed, including *Saccharomyces cerevisiae*, *Caenorhabditis elegans*, and *Strongylocentrotus purpuratus *[43-47]. However, finding and quantifying mammalian networks has been challenging due to the data requirements of current methods. In the present analysis, we have demonstrated the utility of bioinformatics approaches for elucidation of a GRN describing proneural bHLH (Ngn2 and Mash1) transcription factor regulation of murine telencephalon specification.

To develop a robust GRN, we first measured global gene expression patterns in GOF and LOF analyses to identify novel putative target genes for Ngn2 and Mash1, several of which were validated by *in situ *hybridization in slice preparations from mutant and electroporated mouse embryos. Using Bayesian network analysis, we have corroborated 25 literature-based GRN hypotheses by quantifying connections based on the compiled microarray datasets.

As highly conserved long-range enhancers are known to be particularly important for developmentally regulated genes [48-50], we utilized phylogenetic footprinting analyses to identify putative long-range enhancers in several predicted Ngn2 and Mash1 target genes. This approach suggests that no putative co-factor binding sites are specifically associated with conserved Mash1 binding sites in both Mash1 and common target genes, consistent with an instructive and relatively context-independent role for Mash1 in ventral cell fate determination [51]. In addition, the analysis identifies several conserved TFBSs in close proximity to Ngn2 binding sites surrounding the Ngn2 target genes, consistent with previous findings supporting a more cell context-dependent role for Ngn2 in dorsal telencephalic fate specification [51]. In addition, we hypothesized that important regulatory modules that bind transcription factors other than Ngn2 and Mash1 may be present surrounding the identified target genes. Therefore, our second comparative genomics analysis was designed to search for conserved TFBS enriched in target genes, but not necessarily in close proximity to Mash1 or Ngn2 binding sites. Using a novel Bayesian network analysis approach with an informative prior structure, we were able to synthesize the knowledge gained from each of the above experimental and computational approaches to predict connectivity between the novel target genes, co-factors, and co-regulators.

Our resultant GRN predicts that Creb1 and Crebbp are the most likely candidates for a dorsally expressed Ngn2 co-factor regulating cortical targets such as *Neurod6*, *Eomes *and *NeuroD2*. Interestingly, previous analyses have indicated interactions between bHLH transcription factors, Creb, and the Creb binding protein (CBP/P300 complex) in the differentiation of several cell types [52,53], as well as in neurotrophin-mediated expression of *vgf *[54]. Most notably, a conserved *Neurod6 *promoter has recently been shown to contain both Creb and E-box binding sites and is activated via cAMP exposure [55]. Yy1 is also a potential Ngn2 co-factor, yet our microarray data suggest that *Yy1 *mRNA expression is confined to the ventral telencephalon. This finding suggests an inhibitory role for Yy1 in Ngn2-regulated transcription, which is supported by evidence showing Yy1 inhibition of both BMP induced cell differentiation [56] and Notch transcriptional activity [57].

Our GRN also supports a synergistic interaction between Pou-domain containing transcription factors and bHLH proneural proteins in the activation of several target genes. Interestingly, both Pou6f1 and Mash1 have been identified as important mediators of oligodendrocyte development [58,59], and Pou6f1 is thought to synergistically interact with members of the Sox family [60,61]. Moreover, other members of the Pou domain, class 3 family of transcription factors, Brn1 or Brn2, which exhibit similar affinity with Pou6f1 for certain binding sites [62,63] have been experimentally shown to co-regulate several Mash1 targets in the ventral telencephalon [12].

Finally, our analysis predicts connectivity between *Sox9*, *Fzd1*, and *Wnt7b*, which is consistent with a recent report suggesting *Sox9 *regulation through Wnt signaling [64]. Sox9 may serve as an inhibitory factor for several Ngn2 target genes including *Gng2*, *Npdc1*, *Eomes*, *Fzd1*, and *Coro2b*, consistent with the opposing roles of Sox9 and Ngn2 in the specification of glial fate [35,36]. We also show that Mef2a, which has been shown previously to have complex regulatory functions in neuronal differentiation and plasticity [38-40], may co-activate several of the predicted Ngn2 targets, such as *Neurod*, *Ngn1*, *Wnt7b*, *Mgst3*, *Gng2*, *Acpl2*, and *Dusp14*. In addition, our findings suggest that transcriptional regulators downstream of Wnt signaling (Tcf4/Lef1) may bind to regulatory modules that also bind Ngn2, which is consistent with the role of Wnt signaling in the specification of the dorsal forebrain [65-67], and offers a hypothesis in which coordinated Wnt activation and Ngn2 expression act in concert to transcriptionally activate target genes. Interestingly, a recent report suggests that Wnt pathway-initiated neural differentiation, but not proliferation, requires specific interactions between Tcf/β-catenin and Crebbp [68]. Another intriguing aspect of our analysis is the prediction of *Elavl4 *as an important regulator of Ngn2 targets, including *Bhlhb5*, *Robo1*, *Nhlh1*, *Coro2b*, *Sox11*, and *Dll1*, possibly through stabilization of mRNA [69]. It is of note that two related mRNA stabilization genes, *Elavl2 *and *Elavl3*, are also predicted targets of Ngn2.

Our current research identifies important research steps for further refinement of the GRN. The cyclic nature of delayed negative feedback in the Notch pathway is thought to act as a molecular clock regulating the timing of several developmental processes [70]. Future production of robust time-series datasets will allow for application of Bayesian methods that are not limited to the discovery of acyclic networks and linear relationships [71]. Several recent reports have suggested connectivity between Notch and Wnt pathways [27,68,72,73]. For example, one way in which the Notch pathway may regulate Wnt signaling is through Lef protein stabilization by Nrarp activation [27]. However, because no data currently exist that would allow quantitative prediction via direct protein-protein interactions, we are unable to predict this relationship in the current network analysis. Nonetheless, our network analyses of transcriptional regulation offers other hypotheses such as a Tcf/Lef regulation of *Lfng *and *Mfng*, which is consistent with evidence during somitogenesis suggesting a Wnt mediated regulation of *Lfng *expression [74].

One important application of the network analysis is the prediction of the most useful perturbation or chromatin immunoprecipitation experiments for resolving the overall network structure [75]. This is particularly relevant to studies in mammalian species, in which perturbation analyses are much more time and resource intensive. Our network analysis would suggest *Centg3 *and *Elavl4 *are important candidates for perturbation and subsequent global gene expression analysis for further network resolution, as they are both highly connected nodes and little is known about their function in the developing telencephalon. Interestingly, *Centg3 *may protect against neurodegeneration in Polyglutamine diseases [76]. The central role of *Elavl4*, which regulates through mRNA stabilization [69], highlights the importance of moving beyond cis regulatory binding to elucidate network relationships.

## Conclusion

In this manuscript, we have combined a number of different approaches to identify regulatory relationships that are important for mammalian telencephalic development, using existing knowledge from the literature, global gene expression data from GOF and LOF studies, bioinformatics-based sequence analyses, and Bayesian network algorithms. Systems biology methodologies that take into account several data sources, such as that presented here, will aid in more rapid identification and quantification of gene regulation which is useful for discovering critical steps in the progression of normal and perturbed human telencephalon development. Eventually, the linkage of mammalian genetic perturbation network analysis with protein interactions and ultimately phenotypic outcome will become possible, as has been initiated in invertebrate models. The methods developed and applied in this manuscript are a first step towards this broader goal.

## Methods

### Electroporation of mouse embryos

Embryos obtained from CBA/CA X C57Bl/10 crosses were dissected without removing placental membranes at embryonic day E10.5, and were transferred into Tyrodes solution [77]. At this stage *Mash1 *and *Ngn2 *are already expressed in the presumptive basal ganglia and cortex, respectively. The neuroepithelium is thus competent for proneural activity and GOF studies should allow the detection of genes regulated by Mash1 or Ngn2. Embryos were precultured for 2 h into a 'precision incubator' (BTC Engineering, Milton, Cambridge, UK) at 37°C with 65% oxygen in 75% v/v Rat serum + 25% v/v Tyrodes solution and 2 mg/ml of glucose. Both telencephalic vesicles were injected for microarray experiments and with only one vesicle for embryos processed for *in situ *hybridization using a FemtoJet Microinjector (Eppendorf) with 2 μl of a solution containing 3 μg/μl Mash1- or Ngn2-pCAGGS expression vector [78] and 2 μg/μl GFP control vector. Electroporation was performed in Tyrodes solution in a CUY520P20 chamber (Nepagene, Japan) using a BTX Electro Square Porator (Eppendorf), with the following settings: 70 V, five pulses, 50 ms at 1 s intervals. Electroporated embryos were cultivated in Rat serum supplemented with glucose as above, for the indicated time. After 18 h, heart-beating embryos were dissected under a UV binocular microscope and the electroporated (GFP-expressing) tissue was dissected under the microscope and homogenized immediately in 300 μl Trizol (Invitrogen). The choice of the time of collection of the tissue was based on maximal expression of the *Dll1 *promoter-lacZ reporter located in the 0.8 kb distal promoter region described previously [79] (see Figure 2(a)) and on a more detailed analysis by quantitative RT-PCR of the time course of induction of *Dll1 *by Mash1 in P19 cells [12]. This work showed that induction of this direct target of Mash1 becomes detectable 4–7 h after *Mash1 *expression but reaches a plateau only after about 15 h. Therefore, this time of collection maximizes the detection of putative direct targets; however, it does not rule out the possibility of detecting indirect targets as well. Total RNA was extracted following manufacturer recommendations and resuspended in 12 μl of diethylpyrocarbonate (DEPC)-treated water (Ambion). Between 3 and 10 electroporated cortices or basal ganglia were pooled to produce a minimum of 1 μg total RNA. The preparation of probes and hybridization to MG430 2.0 chips were performed following Affymetrix guidelines.

### RNA *in situ *hybridization and immunocytochemistry

Electroporated embryos were washed for 30 min at 4°C in phosphate buffered saline (PBS) 1×, fixed in PFA 4% for 3 h, washed again in PBS 1× and incubated in 15% sucrose phosphate buffer 0.12 M (PB), pH 7.2, overnight at 4°C, incubated in gelatin 7.5%/sucrose 15% PB at 42°C and frozen in isopentane at -40°C. Wild-type and mutant embryos (*Ngn2*-/- and *Mash1*-/-) at stage E12.5 or E13.5 were fixed overnight in 4% paraformaldehyde (PAF) at 4°C, incubated overnight as before. Embryonic sections were performed at 10 μM using a Microm cryostat. *In situ *hybridizations were carried out as described previously [12], with NBT/BCIP or fluorescent substrate in the case of *Gp38/Podoplanin *[80]. Mouse *Elavl4 *(*HUC/D*) polyclonal antibody was used as described previously [12]. All of the *in situ *analyses performed are summarized in Additional file 4. Briefly, eight genes putatively regulated by *Ngn2 *and eight genes regulated by *Mash1 *were selected based on their levels of downregulation and upregulation in the microarray data, as well as their potential involvement in neuronal development (*Mnfg*, *Lnfg*, *Lhx8*, *HuC/D*, *Nscl1*) or expression in the developing nervous system (*Gp38/podoplanin*, *Rhomboid*, *Nrarp*). Two out of eight Ngn2 candidate genes were not detected *in situ *in the cortex of WT embryos and were not tested further. Candidates showing a consistent regulation in *Ngn2 *or *Mash1 *LOF mutant embryos (five out of six expressed genes for Ngn2 and six out of eight genes for Mash1) were further analyzed by *in situ *hybridization on electroporated GOF embryos.

### P19 cell transfection and quantitative RT-PCR

P19 embryonal carcinoma cells are pluripotent cells that specifically differentiate into neurons when induced by retinoic acid or when transfected with *Mash1*, *Ngn1*, or *NeuroD *expressing vectors [81,82]. We seeded 250,000 P19 cells into 21 cm culture dishes in DMEM (Gibco) supplemented with 5% goat serum and incubated overnight at 37°C. Cells were transfected in duplicate with 2 μg *Ngn2*-, *Mash1*-, or empty pCAGGS vectors and 0.1 μg GFP control vector mixed with Lipofectamine 2000 (Invitrogen) following the manufacturer's recommendations. Total RNA extraction was performed in 2 ml Trizol (Invitrogen). RNA pellets were resuspended in 50 μl DEPC treated water (Ambion) and the RNA concentration was determined by spectrophotometry. A total of 2 μg RNA was treated with 10 units DNase I (Invitrogen) and reverse transcribed with Superscript III (Invitrogen). Quantitative PCR was performed in duplicates with SYBR Green (Roche) on a Light cycler apparatus (Roche). A cDNA from hydroxymethylbilane synthase was used as a reference for normalization. Primer sequences are available from the authors upon request.

### Global gene expression analyses

Twenty-eight separate gene expression datasets were used for the identification and quantification of GRNs for forebrain development using either the U74A and U74B or MOE430 2.0 Affymetrix microarray platforms. Analyses of tissues from dorsal and ventral telencephalon from wild-type (*n *= 14), *Ngn1-/-*, *Ngn2-/-*, *Mash1-/-*, *Ngn1-/-*; *Ngn2*-/-, and *Ngn2-/-*; *Mash1-/- *transgenic mice and GOF tissues from mice in which *Ngn2 *or *Mash1 *were electroporated on E10.5 and killed 18 h later (see above) are included in this dataset. Analyses of *Ngn1*, *Ngn2*, and *Mash1 *single and double knockouts were performed with RNA extracted from tissue dissected from E13.5 mice and hybridized to U74A and U74B Affymetrix chips. Basal ganglia from *Mash1 *KO embryos were dissected and processed for RNA trizol extraction as described previously [4]. Microarray data from cortical tissue have been described previously [4]. Two replicates of each control and a single knockout were analyzed, whereas one replicate for each double knockout and control was sampled. Microarray analyses of dorsal and ventral telencephalic tissues from control and GOF mice (E10.5 mice cultured for 18 h) were performed using the Affymetrix MOE430 2.0 chip. Replicates were performed for a total of two dorsal telencephalon controls, three ventral telencephalon controls, and three each of the *Ngn2 *and *Mash1 *GOF mice. Normalization was performed using GC-RMA software for background adjustment using sequence information [83] downloaded from [84]. MOE and U74 probesets were assigned Ensembl IDs based on Ensembl Version 37 and duplicate Ensembl IDs were collapsed within a set by taking the median value. Gene expression ratios used for the subsequent network analyses described below were derived from individual mutant arrays versus a time-matched wild-type control gene expression array. To generate a list of putative target genes we used a 1.3-fold cut-off. We note that the use of a fold change cut-off has been shown to be more reliable than *p*-value or false discovery rate (FDR) cut-offs, in a multicenter large-scale quality control analysis across laboratories and platforms [85]. Furthermore, our target gene lists are generated from two independent fold change cut-offs, from both transgenic and GOF experiments, thereby increasing confidence in the resultant target gene lists. GOF experiments were performed at an earlier stage in neurogenesis (E10.5 and cultured for 18 h) than LOF experiments (E13.5) so that expression of *Mash1 *and *Ngn2 *in the telencephalon is still low and the effect of overexpressing these genes is maximal, while the LOF analysis had been performed at a stage when *Mash1 *and *Ngn2 *expression is high, to maximize the effect of loss of these genes. Based on previous analysis of Mash1 and Ngn2 function in telencephalic development [3,4], we do not expect these genes to have substantially different functions and target genes at these two stages.

### Quantification of networks

The strength of the relationships in GRNs were quantified to calculate the posterior probability distribution for the strength of the linkages based on the fold changes seen in the gene expression datasets [8]. A log-linear function was used to describe relationships between genes:

$$\ln(G_{i})=\alpha_{i}+\sum_{j=1,j\neq i}^{n}I_{ji}\beta_{ji}\ln(G_{j})+\varepsilon_{i}$$

where *α*_*I *_is the level of gene expression independent of the network, *I*_*ji *_is an indicator function (0, 1, -1) if a linkage exists from gene *j *to gene *i*, *β*_*ji *_is the degree to which change in gene *j *will affect change in gene *i*, *G*_*j *_is a variable associated with the relative expression level of gene *j *compared with normal level *j*, *e*_*i *_is the random error in predicted value for gene *i *and *n *is the number of genes in the network.

The posterior distributions for the linkages in each network were derived using Markov chain Monte Carlo (MCMC) sampling methods as described elsewhere [8,34]. For the current analysis, KO and GOF effects on genes are modeled as dedicated parents where the prior for *α *_*i *_is set to zero; all other *α *are assumed to have normal priors. The priors for the *β *are assumed normal with mean zero and variance *σ *= 1. Finally, *e*_*i *_is assumed to be normally distributed with mean zero and variance *σ *_2_, where *σ *_2 _is assumed to have a uniform prior with support defined by the observed data. The MCMC maximum sampling step sizes are 0.05 for the *σ*, 0.08 for the *β*, and 0.05 for the *α*, and 500,000 iterations were performed with decimation of every 10th value. The last 50,000 iterations were used to establish the mean value of *β *_*ji *_and the significance of this value. Statistical significance of the parameter *β *_*ji *_is defined by less than 5% of iterations with *β *_*ji *_≤ 0. To address the specificity of our method, we have permuted the gene labels from the microarray experiments (*n *= 12,357) generating 100 random datasets of gene expression. We then applied these datasets to quantitate the lit-based network to determine the number of times we see significance of these connections from each randomly generated dataset. It should be noted that gene expression correlations across experimental conditions are preserved in this analysis. Software for performing these analyses is available from JMG.

### Identification of potential co-factors for Ngn2 and Mash1

Consensus binding sites for Ngn2 and Mash1 were defined as CANTWG and GCAGSTGK, or CAGSTG, respectively, based in part on [12,28,29] and unpublished data (DSC and FG) as described in Additional file 7. Due to the scale of the bioinformatics method performed for predicting co-factors, we limited our analysis to the sequence surrounding 11 predicted Ngn2 target genes, 14 predicted Mash1 genes, and 6 common target genes based on criterion similar to that used for *in situ *confirmation in that we focused on the most differentially expressed as well as the best candidate genes from the literature (Additional file 8). For each gene we looked at a minimum of 500 kb of sequences in front of (approximately 300 kb) and behind (approximately 200 kb) including UTRs and introns of the gene of interest and surrounding genes that fell within the 500 kb range. We utilized the ECR browser [86] to align human sequence with *Mus musculus, Gallus gallus, Xenopus tropicalis, Fugu rubripes*, and *Danio rerio *[87]. Sometimes no conserved regions were found within our search limits, in which case we removed alignments with the lower vertebrates (*F. rubripes *and *D. rerio*) and only analyzed alignments to *G. gallus *and/or *X. tropicalis *to find conserved non-coding regions. To further refine the alignment, the web-based Mulan program was utilized, which performs a full local multi-sequence alignment that can account for evolutionary reshuffling and inversions using the threaded blockset aligner program [87,88]. From this analysis, evolutionary conserved regions (ECRs) with a minimum length of 100 bp and minimal percentage identity of 70% were defined. Finally, we applied Multitf, which searches across the identified ECRs for conserved TFBSs [88], to search for putative Mash1 (GCAGSTGK or CAGSTG) and Ngn2 (CANWTG) binding sites. In total, 160 conserved Ngn2 and 75 conserved Mash1 binding sites were identified. These sites were distributed over the 500 kb analyzed, although the highest number of sites was found in the 20 kb of sequence surrounding the TSS (Additional file 12). Specifically, 19 Mash1 sites were found surrounding the 11 Ngn2 target genes (average number of sites per gene is 1.7) versus 56 Mash1 sites surrounding the 20 Mash1 and common target genes (average number of sites per gene is 2.8). However, this difference is not statistically significant (*p *= 0.2). Furthermore, Ngn2 binding sites are found just as often in front of Ngn2 targets versus Mash1 and/or common target genes (82 Ngn2 sites were found surrounding the 14 Mash1 targets and 22 Ngn2 sites were found surrounding the 6 common targets versus 58 sites found surrounding the 11 Ngn2 target genes). The similarity between Ngn2 and Mash1 and potential function in central nervous system development suggests Mash1 targets could be regulated by Ngn2 as well, in the telencephalon or other tissues. In addition, we note that Mash1 target genes are not equivalent to a random set of genes when analyzing for enrichment of Ngn2 sites. With regards to enrichment of Ebox sites in our putative target genes, the CONFAC analysis described below allowed us to show that several Ebox matrices were significantly enriched in the sequence surrounding our predicted Ngn2 and Mash1 target genes when compared with 250 randomly selected genes (Additional file 9).

To identify potential co-factors, we searched for all vertebrate TRANSFAC annotated TFBSs within 30 bp upstream and downstream of the putative Ngn2 or Mash1 binding sites. The 30 bp length was based in part on prior research showing active modules containing Pou and bHLH binding sites within 15 bp of each other [12]. We removed those TRANSFAC annotated TFBSs that overlapped considerably with the putative Ngn2 and Mash1 sites including the following TRANSFAC matrices: E12, E2A, Heb, Hen1, Hand1, E47, Ebox, myogenin, NeuroD, Myod, Areb6, Tal1, Lbp1, Ap4, E47, and lmo2com. We also collapsed all similar TRANSFAC matrices that referenced the same family of transcription factors (for example, Pou domain containing factors and SRY domain containing factors) or were for the same transcription factor, but identified in different vertebrate species. TRANSFAC matrices were mapped to current mouse gene identifiers by following the original reference for the matrix found in TRANSFAC through the literature. To identify the most likely co-factors for Ngn2 and Mash1, we performed the Fisher's exact two-sided test with *p *< 0.05 to test for significantly enriched TFBSs in sequence surrounding Ngn2 sites versus Mash1 sites or vice versa. Gene expression from wild-type dorsal and ventral telencephalon tissue was analyzed to predict differential dorsal or ventral expression patterns of the predicted co-factors using a 1.5-fold change cut-off. These were subsequently compared with *in situ *analyses found in online databases (Additional file 10).

### Identification of putative co-regulators of Ngn2 and Mash1 targets

Promoter region sequence (10,000 bp upstream of TSS) from mouse and human orthologs of Ngn2 predicted targets and Mash1 and Mash1/Ngn2 predicted common targets (only those with RefSeq IDs associated with them) was automatically uploaded from the UCSC database via the CONFAC website [89]. CONFAC then identifies conserved TFBSs from the TRANSFAC database version 7.0 in the human and mouse sequence alignments [30]. As part of the CONFAC software, the Mann-Whitney statistical test was then applied to test for enrichment of TFBSs in the given gene lists. We compared each list with a list of 250 randomly picked genes available from the CONFAC website, as well as comparing our Ngn2 list with the Mash1/common targets list and vice versa. We then annotated the resulting lists of enriched TRANSFAC TFBSs as described above. Transcription factors that did not show minimal expression (> 4.5 median intensity) in wild-type microarray datasets were not analyzed further.

### Algorithm-based network structure

The TAO-Gen algorithm identifies the optimal gene regulatory network given a specific gene expression dataset [34]. Briefly, our method utilizes a log-linear model (Equation 1) and MCMC to identify the network that best accounts for the variability seen in the microarray datasets. In order to explore larger networks, the number of possible networks in the search space is restricted. This is accomplished through use of an annealing algorithm that combines aspects of the Metropolis algorithm used for MCMC sampling and a simulated annealing algorithm used for optimizations. The maximum number of parents for any given gene is restricted to three; however, no complexity penalty was used. Based on standard techniques in Bayesian networks [90], we include all network structures within the top 95% of scores based on the maximum likelihood and build a common network that includes those interactions that occur in more than 50% of these network structures. A detailed description, as well as a complete evaluation of this method through statistical simulation studies has been described previously [34]. We have also performed detailed comparisons to another Bayesian network algorithm [35], which was subsequently coded for Matlab [36]. Results are described in detail in Additional file 1.

An informative prior structure was built utilizing several different data sources. The informative prior structure is represented as a matrix with 0 meaning forbidden connection, 1 meaning required connection, and 0.5 meaning no prior information is available. We considered 25 literature-based connections as required connections, based on previous literature data that is consistent with the current microarray data, which are highlighted in the resulting network. Genes whose functions are known and do not include direct transcription factor activity or DNA/RNA binding are forbidden from being parents, with the exception of the signaling molecules Wnt7b and Dll1, which are known initiators of transcription via Wnt/β-catenin and Notch pathways, respectively.

Parents are required to be expressed in the same tissue as children (greater than 4.5 median intensity (log2 base) in wild-type datasets); therefore, solely dorsally expressed genes are not permitted to parent ventrally expressed genes and vice versa. Each TFBS information source is given an informative prior value of 0.1, such that if a TFBS is found in a sequence in front of a given gene, the prior score is raised from 0.5 to 0.6. TFBS data were derived from both comparative genomics analyses described above. Results obtained with and without the prior structure are described in Additional file 1.

## Authors' contributions

JMG, OA, GG, CJP, and FG designed the research. OA, CZ, DSC, and LN performed the experimental research. JMG, FMP, MVS, and JSP performed the computational and bioinformatics research. JMG, OA, CJP, and FG wrote the manuscript. All authors read and approved the final manuscript.

## Supplementary Material

Additional file 1 **Description of the literature-based network shown in **Figure 1. Comparison of the TAO-Gen algorithm with other Bayesian based algorithms. Click here for file

Additional file 2 Expression of the Ngn2 target *NeuroD *analyzed by *in situ *hybridization in the dorsal telencephalon after electroporation of *Ngn2*. Click here for file

Additional file 3 List of predicted targets categorized by GO. Click here for file

Additional file 4 List of targets analyzed using *in situ *hybridization in wild-type and mutant embryos ( *Ngn2-/- *and *Mash1-/-*), and of embryos overexpressing *Ngn2 *or *Mash1*. Click here for file

Additional file 5 Quantitative PCR analysis of predicted Mash1 targets in P19 cells following transfection of a *Mash1 *expression vector. Click here for file

Additional file 6 Quantification of literature-based network structure. Click here for file

Additional file 7 Determination of Ngn2 and Mash1 consensus binding sites. Click here for file

Additional file 8 List of Ngn2, Mash1, and common target genes examined in co-factor/co-regulator analyses. Click here for file

Additional file 9 CONFAC results predicting co-regulators for Ngn2 targets and common targets and enrichment of Ebox sites surrounding Ngn2 and Mash1 target genes, as well as differential gene expression of predicted co-regulators. Click here for file

Additional file 10 Corroboration of predicted co-factors, co-regulators and related proteins via interrogation of online databases. Click here for file

Additional file 11 Matrix of connectivity of algorithm-based gene regulatory network structure for dorsal and ventral telencephalon development. Click here for file

Additional file 12 Histogram of positions of putative Ngn2 and Mash1 binding sites from TSS. Click here for file
