# Supplementary material for: Characterization of the proneural gene regulatory network during mouse telencephalon development
Source: BMC Biol. 2008 Mar 31;6:15. doi: 10.1186/1741-7007-6-15 (PMC2330019; doi:10.1186/1741-7007-6-15)
Supplement: Additional file 1 — Description of the literature-based network shown in Figure 1. Comparison of the TAO-Gen algorithm with other Bayesian based algorithms. [file 1741-7007-6-15-S1.doc]

Description of literature based gene regulatory network

*Ngn1* and *Ngn2* are required to specify early-born glutamatergic neurons (those expressing markers such as *VGLUT2*/*Slc17a6*) that populate Layers V-VI of the developing neocortex and specifically express *Robo1, Etv1*, and *Tbr1* ^1^. *Otx1* is also a marker for deep layer neurons in the developing dorsal telencephalon, however, it is also expressed in progenitor cells ^2^. Based in part on evidence in cranial ganglia, *Ngn1* and *Ngn2* positively regulate *Neurod2, Neurod* and *Nscl1* ^3,4^. *Ngn2* is activated by the Wnt pathway in dorsal forebrain^5,6^, and can subsequently repress subcortical GABAergic neuronal phenotypes through inhibition of *Mash1* ^1^.

Pax6 activates *Ngn2* and *Ngn1*^7,8^, while *Ngn2* may cooperate with *Pax6* to regulate *Ngn1* in the developing dorsal forebrain ^9^. Ngns are thought to activate the glutamatergic phenotype through activation of an intermediate set of bHLH neural differentiation transcription factors, including *Neurod, Neurod6* and *Neurod2* ^10,11^ and the homeobox transcription factor *Eomes* /*Tbr2*, which in turn may regulate *Tbr1,* based on sequential expression patterns^12^. *Id2* is a cortical neuronal marker that may be regulated by *Ngn2*, most likely via downstream bHLH transcription factors, such as the *Neurods*^1,13^. *Pax6* expression is necessary for the differentiation of late-generated neurons that populate Layers II-IV and express *Satb2* and *Pou6f1*/*Oct6*^14,15^.

*Mash1* initiates specification and differentiation of inhibitory neurons in the ventral telencephalon through activation of the homeobox transcription factors *Dlx1* and *Dlx2*, which in turn activate *Dlx5*^16^. *Dlx1* and *Dlx2* are thought to play a role in the activation of *Arx*, an important determinant of GABAergic cell fate^17^. Predicted downstream targets of the Dlxs include *Gad1, Gad2, Slc6a1*/*GABA-T1, Slc32a1*(*GABA/glyT*)^1^.

The neural progenitor population within both the dorsal and ventral forebrain during this period of neurogenesis express high levels of bHLH inhibitory proteins including *Hes1* and *Hes5.* The *Hes* genes are thought to be indirectly activated by proneural bHLHs through transcriptional activation of the Notch ligand, *Dll1*^18^.

Comparing the literature based network to network generated by TAO-Gen and Werhli et al. (2006) Bayesian Network algorithm

The significant connections generated by TAO-Gen using only gene expression changes of the 29 genes in the literature based network is shown below (List 1). The mean posterior probability of the lit-based network structure (as shown in Figure 1 in main text and Additional file 6) is -510 whereas the network generated by TAO-Gen has a mean posterior probability of -763, suggesting the literature based network structure has a higher probability of being the true network given the data. However, we note comparing the literature based posterior likelihood to the posterior likelihood of the best network generated from an algorithm using only the microarray data may not be appropriate, as it is comparing networks generated by very different methods, yet the posterior log likelihood only can assess the ability of the network structure to describe the microarray data at hand.

Comparing TAO-Gen to an alternative Bayesian-based algorithm

We have generated networks using an alternative Bayesian algorithm developed by Geiger and Heckerman (1994)^19^ and coded for Matlab by Werhli et al. (2006) (List 2 using small 29 gene list and List 3 using 86 genes) and have compared results with those generated using TAO-Gen without the informative prior (List 1 and 4). For the Geiger and Heckerman algorithm analysis, we computed the results of 100,000 runs for the 29 lit based gene list and 10,000 runs for the 86 gene list, including all connections which were in at least 10% of the top scoring 5000 or 1000 networks, respectively. It was necessary to limit the number of runs for the 86 gene analysis to 10,000 runs, as computational time was prohibitive. In general, we conclude the algorithms are similar and give consistent results, in that several of the connections found by the Geiger and Heckerman algorithm are also found by TAO-Gen, although the Geiger and Heckerman algorithm does suggest Rhbdl4 is an important parent node, whereas TAO-Gen does not. We note TAO-Gen produces a network with a higher mean posterior log likelihood score (-763 vs. -1070 when just using lit based genes and -1839 vs. -2001 when using 86 gene list).

TAO-Gen predicts a greater number of connections than the Geiger and Heckerman algorithm (34 vs. 19 in lit-based and 105 vs. 36 in the full network). We suggest this difference may be in part due to the required weight placed on the non-informative prior in the Geiger and Heckerman algorithm, which is at least equal to the number of genes looked at. Therefore, this heavily weights zero connections when the dataset is small. Alternatively, within TAO-Gen we have chosen not to implement our complexity penalty in our final analysis. However, when the TAO-Gen complexity penalty is implemented, it behaves in a similar way by limiting the total number of connections found to only those that are most strongly suggested by the data (List 5). Finally, when Werlhi et al. (2006) compared their Bayesian method to pairwise evaluations, they demonstrated Bayesian methods produce more robust results^20^.

Comparing TAO-Gen with and without an informative prior

Running TAO-Gen with no informative prior structure produces very different results (List 4,5 compared with List 6), as connectivity is based solely on the strength of linear relationships in gene expression across the microarrays. Therefore, functionality of the protein product (e.g. whether it binds to DNA), spatially restricted expression patterns, as well as the presence of appropriate binding sites are not taken into account. In this analysis the most highly connected parent nodes were predicted as Arx, Nrarp, Ndpc1, and CyclinG2. From this list, only Arx has known DNA binding capabilities, highlighting the utility of the prior structure as an important tool for discriminating between probable indirect and direct regulators. The posterior log likelihood for the network without an informative prior is -1820, whereas the network generated with an informative prior is -1957. However, it should be noted that the posterior log likelihood only considers how well the network describes the microarray data. It does not consider how well the network describes or predicts previous literature data, phylogenetic footprinting data, etc.

**List 1: TAO-Gen network, no informative prior (29 genes):**

| Arx to Slc6a1 |
| --- |
| Dll1 to Nscl |
| Dlx1 to Gad1 |
| Dlx2 to Dlx1 |
| Dlx2 to Dlx5 |
| Dlx5 to Gad1 |
| Hes1 to Dlx5 |
| Hes5 to Dll1 |
| Hes5 to Hes1 |
| Hes5 to Mash1 |
| Hes5 to Neurog2 |
| Id2 to Tbr1 |
| Neurod2 to Neurod6 |
| Neurod2 to Slc17a6 |
| Neurod4 to Pax6 |
| Neurod4 to Satb2 |
| Neurod4 to Wnt7b |
| Nscl to Dlx2 |
| Nscl to Gad1 |
| Nscl to Mash1 |
| Pax6 to ER81 |
| Pax6 to Robo1 |
| Pax6 to Slc17a7 |
| Pou3f1 to Mash1 |
| Slc17a6 to Neurod1 |
| Slc17a6 to Nscl |
| Slc17a6 to Tbr2 |
| Slc6a1 to Dlx5 |
| Slc6a1 to Neurod6 |
| Slc6a1 to Viaat |
| Tbr1 to Neurod2 |
| Tbr2 to Nscl |
| Viaat to ER81 |
| Viaat to Neurod6 |

**List 2: Geiger and Heckerman network (29 genes):**

| Dlx2 to Dlx1 |
| --- |
| Dlx2 to Dlx5 |
| Dlx2 to Gad1 |
| Dlx2 to Mash1 |
| Dlx2 to Slc32a1 |
| Dlx2 to Slc6a1 |
| Dlx5 to Arx |
| Dlx5 to Gad1 |
| Dlx5 to Mash1 |
| Dlx5 to Slc32a1 |
| Dlx5 to Slc6a1 |
| Etv1 to Arx |
| Etv1 to Slc32a1 |
| Etv1 to Slc6a1 |
| Gad1 to Mash1 |
| Gad1 to Slc32a1 |
| Gad1 to Slc6a1 |
| Neurod2 to Neurod6 |
| Neurod6 to Tbr1 |

**List 3: Geiger and Heckerman network (86 genes):**

| Dlx1 to Gad1 |
| --- |
| Dlx2 to Gad1 |
| Dlx2 to Isl1 |
| Dlx2 to Pax6 |
| Dlx5 to Etv1 |
| Dlx5 to Gad1 |
| Dlx5 to Isl1 |
| Dlx5 to Rgs8 |
| Dlx5 to Slc32a1 |
| Gad1 to Isl1 |
| Hes6 to Mfng |
| Mash1 to Anks1 |
| Mash1 to Isl1 |
| Mash1 to Pdpn |
| Mash1 to Rgs8 |
| Mash1 to Wnt7b |
| Neurod6 to Id2 |
| Neurod6 to Neurod1 |
| Neurod6 to Neurod2 |
| Neurod6 to Snca |
| Rgs8 to Gadd45g |
| Rhbdl4 to Acpl2 |
| Rhbdl4 to Atp9a |
| Rhbdl4 to Bai2 |
| Rhbdl4 to Bhlhb6 |
| Rhbdl4 to Btg2 |
| Rhbdl4 to Dll1 |
| Rhbdl4 to Elavl4 |
| Rhbdl4 to Hes5 |
| Rhbdl4 to Igfs8 |
| Rhbdl4 to Ly6e |
| Rhbdl4 to Mfng |
| Rhbdl4 to Nrarp |
| Rhbdl4 to Rarb |
| Rhbdl4 to Rgs8 |
| Rhbdl4 to Wnt7b |

**List 4: TAO-Gen, no informative prior (86 genes):**

| Acpl2 to Dlx1 |
| --- |
| Arx to Crabp1 |
| Arx to ephrinA4 |
| Arx to Rgs8 |
| Arx to Slc6a1 |
| Arx to Viaat |
| Btg2 to Neurod6 |
| centaurin to Ly6e |
| crebbp to Rgs8 |
| cyclinG2 to Elavl4 |
| cyclinG2 to Gng2 |
| cyclinG2 to Hes1 |
| cyclinG2 to Nfya |
| cyclinG2 to Nrarp |
| cyclinG2 to Sox11 |
| Dix1 to Robo1 |
| Dix1 to Stxbp1 |
| Dll1 to Gadd45g |
| Dll1 to Mash1 |
| Dlx1 to Dlx2 |
| Dlx2 to Mash1 |
| Dlx5 to Dlx1 |
| Dlx5 to Gad1 |
| e12 to egr1 |
| e12 to Wnt7b |
| E2F1 to Gca |
| E2F1 to Nrarp |
| Elavl4 to Rhbdl4 |
| ephrinA4 to Dusp14 |
| Fzd1 to ephrinA4 |
| Gap43 to Acpl2 |
| Gap43 to Ly6e |
| Gap43 to Nscl |
| Gap43 to Snca |
| heb to egr1 |
| heb to Gad2 |
| Hes1 to Hes5 |
| Hes5 to Dll1 |
| Hes5 to Hes6 |
| Hes5 to Pdpn |
| Hes6 to Neurog2 |
| Hes6 to Rgs8 |
| Id2 to Gadd45g |
| Id2 to Nuak1 |
| Id2 to Tbr1 |
| Isl1 to Anks1 |
| lef1 to Etv1 |
| lef1 to Neurod2 |
| lef1 to Pax6 |
| Lhx8 to Mash1 |
| Ly6e to Bai2 |
| Ly6e to Fzd1 |
| Ly6e to Neurod6 |
| Ly6e to Wnt7b |
| Mfng to Hes5 |
| Mfng to Igsf8 |
| Mgst3 to Neurog2 |
| Neurod2 to Slc17a6 |
| Nfya to Viaat |
| Nfyb to centaurin |
| Nfyb to Dusp14 |
| Npdc1 to Atp9a |
| Npdc1 to centaurin |
| Npdc1 to Dix1 |
| Npdc1 to Gng2 |
| Npdc1 to Mgst3 |
| Nrarp to Anks1 |
| Nrarp to Btg2 |
| Nrarp to Crabp1 |
| Nrarp to Lfng |
| Nrarp to Rarb |
| Nrarp to Rhbdl4 |
| Nscl to Dlx2 |
| Nuak1 to Gad1 |
| Nuak1 to Neurod2 |
| Nuak1 to Nscl |
| Nuak1 to Satb2 |
| Otx1 to Rhbdl4 |
| Pax6 to Arx |
| Pax6 to Nscl |
| Pax6 to Pou2f1 |
| Pou2f1 to Nuak1 |
| Pou3f1 to Lhx8 |
| Pou3f1 to Pdpn |
| Pou6f1 to Slc6a1 |
| Rhbdl4 to Mfng |
| Satb2 to Gad1 |
| Slc17a6 to Neurod1 |
| Slc17a6 to Tbr2 |
| Slc6a1 to Crabp1 |
| Slc6a1 to Etv1 |
| Snca to Id2 |
| Sox11 to Bai2 |
| Sox11 to Bhlhb5 |
| Sox11 to Elavl4 |
| sp1 to Neurod6 |
| Stxbp1 to Coro2b |
| Stxbp1 to Gap43 |
| Tbr1 to Neurod2 |
| Tbr2 to Glcci1 |
| tcf4 to Lfng |
| TEF to Arx |
| TEF to Gca |
| Viaat to Glcci1 |
| Wnt7b to Bhlhb5 |

**List 5: TAO-Gen, no informative prior, complexity penalty=6** (86 genes):

| Arx to Rgs8 |
| --- |
| Arx to Slc6a1 |
| cyclinG2 to Elavl4 |
| Dix1 to Stxbp1 |
| Dlx1 to Dlx5 |
| Dlx2 to Dlx1 |
| Dlx5 to Etv1 |
| Dlx5 to Gad1 |
| Elavl4 to Bhlhb5 |
| ephrinA4 to Dusp14 |
| Gap43 to Acpl2 |
| Gap43 to Ly6e |
| Gap43 to Snca |
| Glcci1 to Viaat |
| Hes5 to Dll1 |
| Hes5 to Hes6 |
| Hes5 to Pdpn |
| Hes6 to Rgs8 |
| Id2 to Tbr1 |
| Isl1 to Anks1 |
| Lfng to Gadd45g |
| Ly6e to Wnt7b |
| Mfng to Hes5 |
| Mfng to Igsf8 |
| Mfng to Rhbdl4 |
| Neurod1 to Slc17a6 |
| Neurod2 to Neurod6 |
| Neurod2 to Nuak1 |
| Npdc1 to Atp9a |
| Npdc1 to Dix1 |
| Nrarp to Btg2 |
| Nrarp to Mfng |
| Nrarp to Rarb |
| Nscl to Dlx2 |
| Nscl to Mash1 |
| Nuak1 to Nscl |
| Pdpn to Mash1 |
| Slc17a6 to Neurod2 |
| Slc6a1 to Crabp1 |
| Stxbp1 to Coro2b |
| Stxbp1 to Gap43 |

**List 6: TAO-Gen, with informative prior (86 genes):**

| Arx to Bai2 |
| --- |
| Arx to Crabp1 |
| Arx to Fzd1 |
| Arx to Isl1 |
| Arx to Ly6e |
| Arx to Neurod1 |
| Arx to Neurod6 |
| Arx to Neurog1 |
| Arx to Slc17a6 |
| Arx to sp1 |
| Arx to Wnt7b |
| Arx to yy1 |
| Bai2 to Rgs8 |
| creb to Acpl2 |
| creb to Btg2 |
| creb to Lfng |
| creb to Otx1 |
| creb to Robo1 |
| creb to Stxbp1 |
| crebbp to Atp9a |
| crebbp to Lfng |
| crebbp to Mfng |
| Dix1 to Atp9a |
| Dix1 to Ly6e |
| Dix1 to Rarb |
| Dll1 to Gadd45g |
| Dll1 to Hes1 |
| Dll1 to Hes5 |
| Dll1 to Lfng |
| Dll1 to Nrarp |
| Dll1 to Pdpn |
| Dll1 to Rhbdl4 |
| Dlx1 to Dlx2 |
| Dlx1 to Dlx5 |
| Dlx1 to Nfyb |
| Dlx2 to Dlx5 |
| Dlx5 to Gad1 |
| Dlx5 to Gad2 |
| Dlx5 to Slc6a1 |
| Dlx5 to Viaat |
| E2F1 to Dlx1 |
| E2F1 to Gca |
| E2F1 to Gng2 |
| E2F1 to Hes1 |
| E2F1 to Hes5 |
| E2F1 to Viaat |
| egr1 to Neurod6 |
| egr1 to Otx1 |
| egr1 to Pax6 |
| egr1 to sp1 |
| egr1 to Tbr2 |
| egr1 to TEF |
| Elavl4 to Btg2 |
| Elavl4 to Dix1 |
| Elavl4 to Dll1 |
| Elavl4 to Dlx2 |
| Elavl4 to Gad1 |
| Elavl4 to Gng2 |
| Elavl4 to Nscl |
| Elavl4 to Robo1 |
| Elavl4 to Stxbp1 |
| Etv1 to Dlx5 |
| heb to Anks1 |
| heb to Hes6 |
| heb to Npdc1 |
| Hes5 to Hes6 |
| Hes5 to Npdc1 |
| Hes6 to Bai2 |
| Hes6 to Mfng |
| Hes6 to Rgs8 |
| Id2 to Gap43 |
| Id2 to Rarb |
| Id2 to Tbr1 |
| Isl1 to Anks1 |
| lef1 to Anks1 |
| lef1 to Bai2 |
| lef1 to Glcci1 |
| lef1 to Hes6 |
| lef1 to Pdpn |
| Lhx8 to Gadd45g |
| Mash1 to Dll1 |
| Mash1 to Dlx1 |
| Mash1 to Dlx2 |
| mef2a to Dll1 |
| mef2a to Dusp14 |
| mef2a to Elavl4 |
| mef2a to Hes1 |
| mef2a to Id2 |
| mef2a to Nfyb |
| mef2a to Pou2f1 |
| Neurod1 to Id2 |
| Neurod1 to Nscl |
| Neurod1 to Robo1 |
| Neurod1 to Slc17a6 |
| Neurod2 to Neurod6 |
| Neurog1 to Neurod1 |
| Neurog1 to Neurod2 |
| Neurog1 to Nscl |
| Neurog2 to Ly6e |
| Neurog2 to Mash1 |
| Neurog2 to Neurod1 |
| Neurog2 to Neurog1 |
| Neurog2 to Tbr2 |
| Nfya to Elavl4 |
| Nfya to Pou3f1 |
| Nfyb to Coro2b |
| Nfyb to e12 |
| Nfyb to Etv1 |
| Nfyb to Glcci1 |
| Nfyb to Rgs8 |
| Nfyb to Slc6a1 |
| Nscl to Bhlhb5 |
| Nscl to yy1 |
| Pax6 to Pou2f1 |
| Pax6 to Satb2 |
| Pax6 to Slc17a6 |
| Pax6 to Wnt7b |
| Pou2f1 to Crabp1 |
| Pou2f1 to Dlx1 |
| Pou2f1 to Etv1 |
| Pou2f1 to Gadd45g |
| Pou2f1 to Gap43 |
| Pou2f1 to Mfng |
| Pou2f1 to Neurod2 |
| Pou3f1 to Isl1 |
| Pou3f1 to Lhx8 |
| Pou6f1 to Crabp1 |
| Pou6f1 to Gca |
| Pou6f1 to Gng2 |
| Pou6f1 to Isl1 |
| Pou6f1 to Otx1 |
| Pou6f1 to Tbr1 |
| Satb2 to Igsf8 |
| Sox11 to Acpl2 |
| Sox11 to Atp9a |
| Sox11 to Dusp14 |
| Sox11 to ephrinA4 |
| Sox11 to Mgst3 |
| Sox11 to Snca |
| Sox11 to sp1 |
| sox9 to Btg2 |
| sox9 to Dusp14 |
| sox9 to Fzd1 |
| sox9 to Gad1 |
| sox9 to Gca |
| sox9 to Hes1 |
| sox9 to Mgst3 |
| sox9 to Pdpn |
| sox9 to Rarb |
| sox9 to Satb2 |
| sox9 to Snca |
| sox9 to Tbr2 |
| sox9 to Wnt7b |
| sox9 to yy1 |
| Stxbp1 to Rhbdl4 |
| Tbr1 to Neurod2 |
| Tbr2 to cyclinG2 |
| Tbr2 to Tbr1 |
| tcf4 to E2F1 |
| tcf4 to mef2a |
| tcf4 to Nfya |
| tcf4 to Nfyb |
| tcf4 to Npdc1 |
| tcf4 to Pou2f1 |
| tcf4 to Pou6f1 |
| tcf4 to Slc6a1 |
| tcf4 to sox9 |
| tcf4 to Viaat |
| TEF to Acpl2 |
| TEF to Etv1 |
| TEF to Fzd1 |
| Wnt7b to Coro2b |
| Wnt7b to Igsf8 |
| Wnt7b to Sox11 |
| Wnt7b to Stxbp1 |

**Citation:**

1. Schuurmans C, Armant O, Nieto M, et al. Sequential phases of cortical specification involve Neurogenin-dependent and -independent pathways. *Embo Journal* 2004;**23**(14)**:**2892-2902.

2. Simeone A, Puelles E, Acampora D. The Otx family. *Current Opinion in Genetics & Development* 2002;**12**(4)**:**409-415.

3. Ma QF, Chen ZF, Barrantes ID, de la Pompa JL, Anderson DJ. neurogenin1 is essential for the determination of neuronal precursors for proximal cranial sensory ganglia. *Neuron* 1998;**20**(3)**:**469-482.

4. Fode C, Gradwohl G, Morin X, et al. The bHLH protein NEUROGENIN 2 is a determination factor for epibranchial placode-derived sensory neurons. *Neuron* 1998;**20**(3)**:**483-494.

5. Gunhaga L, Marklund M, Sjodal M, Hsieh JC, Jessell TM, Edlund T. Specification of dorsal telencephalic character by sequential Wnt and FGF signaling. *Nature Neuroscience* 2003;**6**(7)**:**701-7.

6. Hirabayashi Y, Gotoh Y. Stage-dependent fate determination of neural precursor cells in mouse forebrain. *Neuroscience Research* 2005;**51**(4)**:**331-336.

7. Blader P, Lam CS, Rastegar S, et al. Conserved and acquired features of neurogenin1 regulation. *Development* 2004;**131**(22)**:**5627-5637.

8. Scardigli R, Baumer N, Gruss P, Guillemot F, Le Roux I. Direct and concentration-dependent regulation of the proneural gene Neurogenin2 by Pax6. *Development* 2003;**130**(14)**:**3269-3281.

9. Fode C, Ma Q, Casarosa S, Ang SL, Anderson DJ, Guillemot F. A role for neural determination genes in specifying the dorsoventral identity of telencephalic neurons. *Genes & Development* 2000;**14**(1)**:**67-80.

10. Lin CH, Stoeck J, Ravanpay AC, Guillemot F, Tapscott SJ, Olson JM. Regulation of neuroD2 expression in mouse brain. *Developmental Biology* 2004;**265**(1)**:**234-245.

11. Mattar PA, Britz O, Johannes C, et al. A screen for novel genes downstream of neurogenin 2 in the developing neocortex. *Developmental Biology* 2004;**271**(2)**:**581-581.

12. Englund C, Fink A, Lau C, et al. Pax6, Tbr2, and Tbr1 are expressed sequentially by radial glia, intermediate progenitor cells, and postmitotic neurons in developing neocortex. *Journal of Neuroscience* 2005;**25**(1)**:**247-251.

13. Neuman K, Nornes HO, Neuman T. Helix-Loop-Helix Transcription Factors Regulate Id2 Gene Promoter Activity. *Febs Letters* 1995;**374**(2)**:**279-283.

14. Britanova O, Akopov S, Lukyanov S, Gruss P, Tarabykin V. Novel transcription factor Satb2 interacts with matrix attachment region DNA elements in a tissue-specific manner and demonstrates cell-type-dependent expression in the developing mouse CNS. *European Journal of Neuroscience* 2005;**21**(3)**:**658-668.

15. Holm PC, Mader MT, Haubst N, Wizenmann A, Sigvardsson M, Gotz M. Loss- and gain-of-function analyses reveal targets of Pax6 in the developing mouse telencephalon. *Molecular and Cellular Neuroscience* 2007;**34**(1)**:**99-119.

16. Fode C, Ma QF, Casarosa S, Ang SL, Anderson DJ, Guillemot F. A role for neural determination genes in specifying the dorsoventral identity of telencephalic neurons. *Genes & Development* 2000;**14**(1)**:**67-80.

17. Cobos I, Broccoli V, Rubenstein JLR. The vertebrate ortholog of Aristaless is regulated by Dlx genes in the developing forebrain. *Journal of Comparative Neurology* 2005;**483**(3)**:**292-303.

18. Ohtsuka T, Sakamoto M, Guillemot F, Kageyama R. Roles of the basic helix-loop-helix genes Hes1 and Hes5 in expansion of neural stem cells of the developing brain. *Journal of Biological Chemistry* 2001;**276**(32)**:**30467-30474.

19. Geiger D, Heckerman D. Learning Gaussian networks. *Proceedings of the Tenth Conference on Uncertainty in Artifical Intelligence* 1994**:**pp.235-243.

20. Werhli AV, Grzegorczyk M, Husmeier D. Comparative evaluation of reverse engineering gene regulatory networks with relevance networks, graphical gaussian models and bayesian networks. *Bioinformatics* 2006;**22**(20)**:**2523-2531.
