# Supplementary material for: Characterization of the proneural gene regulatory network during mouse telencephalon development
Source: BMC Biol. 2008 Mar 31;6:15. doi: 10.1186/1741-7007-6-15 (PMC2330019; doi:10.1186/1741-7007-6-15)
Supplement: Additional file 2 — Expression of the Ngn2 target NeuroD analyzed by in situ hybridization in the dorsal telencephalon after electroporation of Ngn2. [file 1741-7007-6-15-S2.pdf]

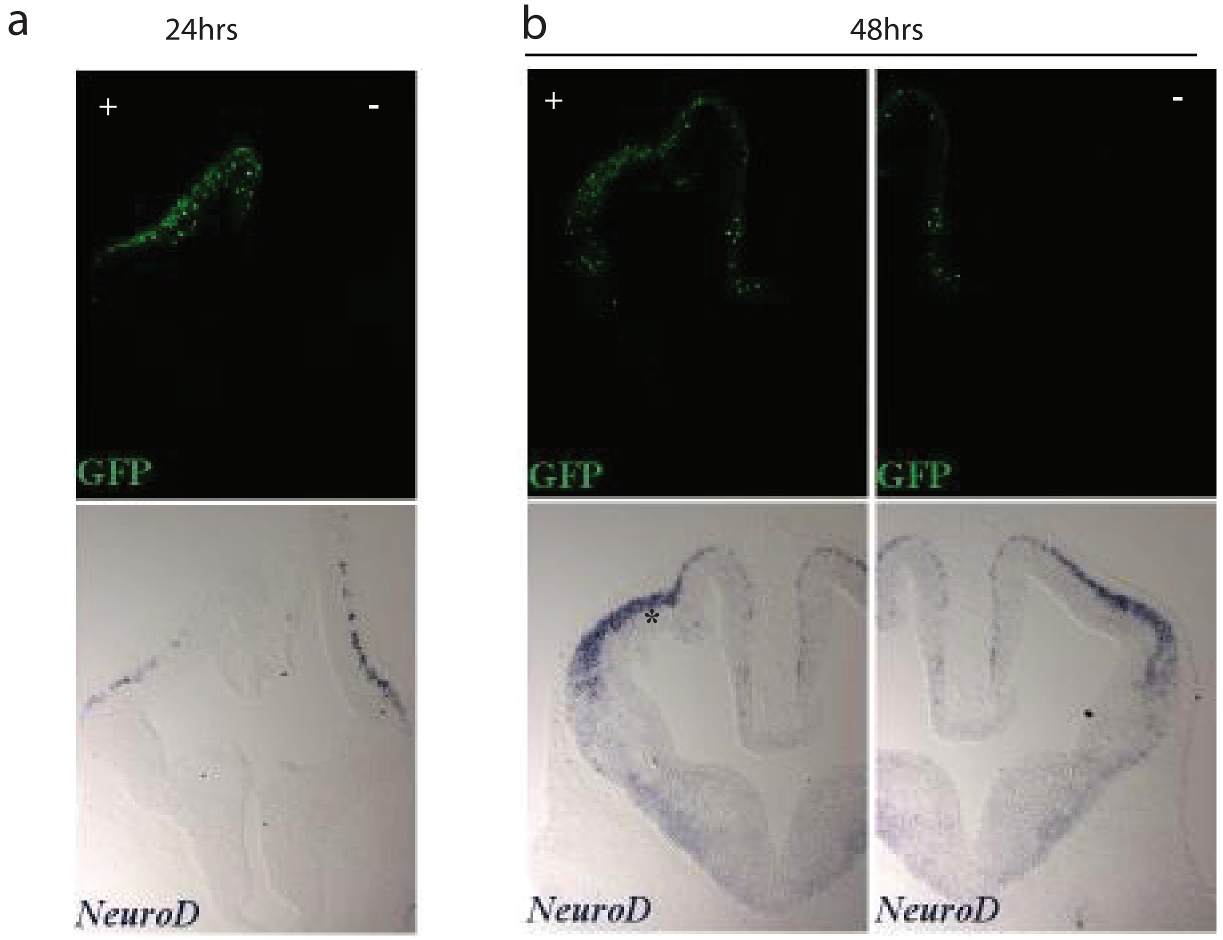

Additional file 2. Expression of the Ngn2 target NeuroD analyzed by in situ hybridization in the dorsal telencephalon. Embryos were electroporated with a Ngn2 expressing vector and cultivated for 24hrs (a) or 48hrs (b). NeuroD upregulation is only detected 48hrs after Ngn2 electroporation. The GFP staining for two different embryos is also shown.
