# Supplementary material for: Characterization of the proneural gene regulatory network during mouse telencephalon development
Source: BMC Biol. 2008 Mar 31;6:15. doi: 10.1186/1741-7007-6-15 (PMC2330019; doi:10.1186/1741-7007-6-15)
Supplement: Additional file 3 — List of predicted targets categorized by GO. [file 1741-7007-6-15-S3.pdf]

Ngn2 targets

Ensembl Transcript ID	Description	Gene Ontology	ratio Ngn2GOF	Mash1-/-;Ngn2-/-
ENSMUST00000035499	neighbor of Punc E11	cell adhesion	1.4	-1.6
ENSMUST00000029674	ephrin A4	cell adhesion	2.6	-1.6
ENSMUST00000059287	Eph receptor B2	cell adhesion	1.3	-1.5
ENSMUST00000064405	Eph receptor A3	cell adhesion	1.7	-1.4
ENSMUST00000020942	Neuronal cell adhesion molecule precursor (Nr-CAM)	cell adhesion	1.6	-2.6
ENSMUST00000020692	B-cell translocation gene 2, anti-proliferative	cell cycle	2.9	-1.4
ENSMUST00000037287	cyclin-dependent kinase inhibitor 1C (P57)	cell cycle	1.4	-3.2
ENSMUST00000031331	cyclin G2	cell cycle	1.7	-1.5
ENSMUST00000030814	cyclin-dependent kinase 5	cell cycle	2.0	-1.5
ENSMUST00000029865	transformation related protein 53 inducible nuclear protein 1	cell death	2.0	-2.8
ENSMUST00000034044	caspase 3	cell death	3.3	-1.3
ENSMUST00000029060	ATPase, class II, type 9A	cellular physiological process	1.8	-2.6
ENSMUST00000021329	golgi SNAP receptor complex member 2	cellular physiological process	1.8	-1.3
ENSMUST00000035861	peroxisome biogenesis factor 5	cellular physiological process	2.7	-1.3
ENSMUST00000052076	ATPase, H+ transporting, V1 subunit G isoform 1	cellular physiological process	1.3	-1.3
ENSMUST00000080882	ATPase, Na+/K+ transporting, alpha 3 polypeptide	cellular physiological process	1.3	-4.0
ENSMUST00000040603	amylo-1,6-glucosidase, 4-alpha-glucanotransferase	cellular physiological process	1.5	-1.4
ENSMUST00000059115	mitochondrial tumor suppressor 1	cellular physiological process	1.4	-1.3
ENSMUST00000052897	thymosin, beta 10 [Source:MarkerSymbol;Acc:MGI:109146]	cytoskeleton component	1.6	-1.4
ENSMUST00000041623	ectodermal-neural cortex 1	cytoskeleton component	1.3	-1.8
ENSMUST00000069284	FYVE, RhoGEF and PH domain containing 4	cytoskeleton component	2.0	-1.6
ENSMUST00000021950	drebrin 1 [Source:MarkerSymbol;Acc:MGI:1931838]	cytoskeleton component	1.8	-1.5
ENSMUST00000010349	Raver1	cytoskeleton component	1.7	-1.3
ENSMUST00000048043	coronin, actin binding protein, 2B [Source:MarkerSymbol;Acc:MGI:2444283]	cytoskeleton component	1.7	-9.1
ENSMUST00000070478	syndecan 3 [Source:MarkerSymbol;Acc:MGI:1349163]	cytoskeleton component	1.4	-1.8
ENSMUST00000092887	myosin XVIIIa	cytoskeleton component	1.3	-1.4
ENSMUST00000003453	phospholipase D family, member 3 [Source:MarkerSymbol;Acc:MGI:1333782]	enzyme	1.7	-1.8
ENSMUST00000007255	dimethylarginine dimethylaminohydrolase 2 [Source:MarkerSymbol;Acc:MGI:1859016]	enzyme	1.5	-1.5
ENSMUST00000029423	serine (or cysteine) peptidase inhibitor, clade I, member 1	enzyme	2.2	-6.0
ENSMUST00000005651	P450 (cytochrome) oxidoreductase [Source:MarkerSymbol;Acc:MGI:97744]	enzyme	1.4	-1.3
ENSMUST00000055808	3-monooxygenase/tryptophan 5-monooxygenase activation protein, gamma polypeptide [Source:MarkerSymbol;Acc:MGI:108109]	enzyme	1.6	-1.6
ENSMUST00000023601	beta galactoside alpha 2,6 sialyltransferase 1 [Source:MarkerSymbol;Acc:MGI:108470]	enzyme	1.3	-1.4
ENSMUST00000005714	ubiquitin-conjugating enzyme E2M (UBC12 homolog, yeast) [Source:MarkerSymbol;Acc:MGI:108278]	enzyme	1.4	-1.3
ENSMUST00000086868	glycerol-3-phosphate acyltransferase, mitochondrial [Source:MarkerSymbol;Acc:MGI:109162]	enzyme	1.4	-1.3
ENSMUST00000028005	microsomal glutathione S-transferase 3	enzyme	1.4	-1.9
ENSMUST00000071776	cysteinyl-tRNA synthetase	enzyme	2.6	-1.4
ENSMUST00000027488	calpain 10 [Source:MarkerSymbol;Acc:MGI:1344392]	enzyme	1.5	-1.8
ENSMUST00000031399	phosphoserine phosphatase [Source:MarkerSymbol;Acc:MGI:97788]	kinase/phosphatase	1.4	-1.4
ENSMUST00000018545	myotubularin related protein 4 [Source:MarkerSymbol;Acc:MGI:2180699]	kinase/phosphatase	1.7	-1.7
ENSMUST00000093394	LIM motif-containing protein kinase 2 [Source:MarkerSymbol;Acc:MGI:1197517]	kinase/phosphatase	2.1	-1.5
ENSMUST00000025270	RIO kinase 3 (yeast)	kinase/phosphatase	1.5	-1.3
ENSMUST00000002487	B-Raf proto-oncogene serine/threonine-protein kinase	kinase/phosphatase	1.8	-1.4
ENSMUST00000077115	protein tyrosine phosphatase, non-receptor type 15	kinase/phosphatase	2.0	-4.8
ENSMUST00000022212	polo-like kinase 2 (Drosophila) [Source:MarkerSymbol;Acc:MGI:1099790]	kinase/phosphatase	1.6	-2.6
ENSMUST00000033930	dual specificity phosphatase 4	kinase/phosphatase	1.4	-8.8
ENSMUST00000057684	PTK2 protein tyrosine kinase 2	kinase/phosphatase	1.8	-1.4
ENSMUST00000059733	dual specificity phosphatase 14 [Source:MarkerSymbol;Acc:MGI:1927168]	kinase/phosphatase	1.9	-2.3
ENSMUST00000041965	Cdc42 binding protein kinase beta [Source:MarkerSymbol;Acc:MGI:2136459]	kinase/phosphatase	1.4	-1.3
ENSMUST00000020220	ZNUAK family, SNF1-like kinase, 1	kinase/phosphatase	1.8	-3.2
ENSMUST00000054996	growth associated protein 43	neuronal physiology, axogenesis	1.5	-3.0
ENSMUST00000031064	dihydropyrimidinase-like 5 [Source:MarkerSymbol;Acc:MGI:1929772]	neuronal physiology, axone guidance	1.8	-1.9
ENSMUST00000000199	frequenin homolog (Drosophila) [Source:MarkerSymbol;Acc:MGI:109166]	neuronal physiology, regulation of syr	1.9	-1.8
ENSMUST00000035058	chondroitin sulfate proteoglycan 5 [Source:MarkerSymbol;Acc:MGI:1352747]	neuronal physiology, synaptic transmi	1.3	-1.5
ENSMUST00000060455	cyclin D-type binding-protein 1	protein binding	1.7	-1.3
ENSMUST00000080103	B-cell leukemia/lymphoma 2	protein binding	2.2	-1.7
ENSMUST00000081165	amyloid beta (A4) precursor protein-binding, family B, member 1 [Source:MarkerSymbol;Acc:MGI:107765]	protein binding	2.0	-1.4
ENSMUST00000080803	olfactomedin 1 [Source:MarkerSymbol;Acc:MGI:1860437]	protein binding	1.5	-1.8
ENSMUST00000079924	BTB (POZ) domain containing 9 [Source:MarkerSymbol;Acc:MGI:1916625]	protein binding	1.7	-1.5
ENSMUST00000030588	polyhomeotic-like 2 (Drosophila)	protein binding	1.5	-1.4
ENSMUST00000008999	histone deacetylase 5 [Source:MarkerSymbol;Acc:MGI:1333784]	regulation of transcription	1.3	-2.1
ENSMUST00000026120	basic helix-loop-helix domain containing, class B5	regulation of transcription	3.5	-1.7
ENSMUST00000055001	Kruppel-like factor 7 (ubiquitous)	regulation of transcription	1.3	-2.3
ENSMUST00000059794	nescient helix loop helix 1	regulation of transcription	1.7	-18.0
ENSMUST00000032551	zinc finger protein interacting with K protein 1	regulation of transcription	2.7	-1.4
ENSMUST00000020974	inhibitor of DNA binding 2 [Source:MarkerSymbol;Acc:MGI:96397]	regulation of transcription	2.0	-2.1

ENSMUST00000024123	centaurin, gamma 3 [Source:MarkerSymbol;Acc:MGI:2183446]	regulation of transcription	2.4	-2.3
ENSMUST00000017945	MAX-like protein X [Source:MarkerSymbol;Acc:MGI:108398]	regulation of transcription	1.5	-1.8
ENSMUST00000006761	CBFA2T1 identified gene homolog (human) [Source:MarkerSymbol;Acc:MGI:104793]	regulation of transcription	1.3	-1.9
ENSMUST00000075448	nuclear factor I/A [Source:MarkerSymbol;Acc:MGI:108056]	regulation of transcription	1.4	-1.8
ENSMUST00000028139	cofactor required for Sp1 transcriptional activation, subunit 8	regulation of transcription	1.9	-1.4
ENSMUST00000030407	lung carcinoma myc related oncogene 1	regulation of transcription	1.4	-1.3
ENSMUST00000079063	SRY-box containing gene 11	regulation of transcription	1.7	-1.6
ENSMUST00000020129	kit ligand [Source:MarkerSymbol;Acc:MGI:96974]	regulation of transcription	1.3	-1.3
ENSMUST00000086851	hairy and enhancer of split 6 (Drosophila)	regulation of transcription	6.0	-2.9
ENSMUST00000049621	hairy and enhancer of split 5 (Drosophila)	regulation of transcription	1.5	-20.8
ENSMUST00000044455	zinc finger protein 451 [Source:MarkerSymbol;Acc:MGI:2137896]	regulation of transcription	1.4	-1.7
ENSMUST00000077225	zinc finger protein 238 [Source:MarkerSymbol;Acc:MGI:1353609]	regulation of transcription	1.3	-1.9
ENSMUST00000048145	RNA binding motif protein 9	RNA binding	2.2	-2.7
ENSMUST00000028251	RNA binding motif protein 18	RNA binding	1.7	-1.3
ENSMUST00000008633	ELAV-like 2 (Hu antigen B)	RNA binding	1.4	-1.6
ENSMUST00000024260	poly(rC) binding protein 4 [Source:MarkerSymbol;Acc:MGI:1890471]	RNA binding	1.8	-1.6
ENSMUST00000003501	ELAV (embryonic lethal, abnormal vision, Drosophila)-like 3 (Hu antigen C) [Source:MarkerSymbol;Acc:MGI:109157]	RNA binding	1.3	-2.7
ENSMUST00000001386	ELAV-like 4 (Hu antigen D)	RNA binding	1.9	-2.7
ENSMUST00000020537	neuron specific gene family member 2 [Source:MarkerSymbol;Acc:MGI:1202070]	signal transduction/Receptor	1.4	-2.4
ENSMUST00000092470	CDC42 effector protein (Rho GTPase binding) 4 [Source:MarkerSymbol;Acc:MGI:1929760]	signal transduction/Receptor	1.9	-1.4
ENSMUST00000023225	glutamate receptor, ionotropic, N-methyl D-asparate-associated protein 1 (glutamate binding) [Source:MarkerSymbol;Acc:MGI:1913418]	signal transduction/Receptor	1.4	-1.7
ENSMUST00000025805	cornichon homolog 2 (Drosophila)	signal transduction/Receptor	1.7	-2.9
ENSMUST00000065587	chemokine orphan receptor 1	signal transduction/Receptor	1.8	-5.1
ENSMUST00000034296	phosphatidylinositol 3-kinase, regulatory subunit, polypeptide 2 (p85 beta) [Source:MarkerSymbol;Acc:MGI:1098772]	signal transduction/Receptor	1.3	-1.6
ENSMUST00000062801	membrane protein, palmitoylated 3 (MAGUK p55 subfamily member 3) [Source:MarkerSymbol;Acc:MGI:1328354]	signal transduction/Receptor	1.3	-1.7
ENSMUST00000004326	plexin A3 [Source:MarkerSymbol;Acc:MGI:107683]	signal transduction/Receptor	2.9	-1.5
ENSMUST00000069557	MAD homolog 5 (Drosophila) [Source:MarkerSymbol;Acc:MGI:1328787]	signal transduction/Receptor	3.9	-1.4
ENSMUST00000093272	G protein-coupled receptor 56 [Source:MarkerSymbol;Acc:MGI:1340051]	signal transduction/Receptor	1.3	-1.4
ENSMUST00000067591	somatostatin receptor 2	signal transduction/Receptor	1.9	-2.4
ENSMUST00000039431	neurotrophic tyrosine kinase, receptor, type 3	signal transduction/Receptor	2.4	-2.6
ENSMUST00000068958	CDC42 effector protein (Rho GTPase binding) 3	signal transduction/Receptor	1.6	-1.4
ENSMUST00000001347	Rho family GTPase 2	signal transduction/Receptor	2.1	-4.5
ENSMUST00000025279	Niemann Pick type C1	signal transduction/Receptor	1.6	-1.4
ENSMUST00000049126	delta/notch-like EGF-related receptor	signal transduction/Receptor	1.4	-3.0
ENSMUST00000026826	Rab40c, member RAS oncogene family [Source:MarkerSymbol;Acc:MGI:2183454]	signal transduction/Receptor	1.5	-1.6
ENSMUST00000037048	monocyte to macrophage differentiation-associated 2 [Source:MarkerSymbol;Acc:MGI:1922354]	signal transduction/Receptor	1.4	-6.1
ENSMUST00000080421	mitogen activated protein kinase 8 interacting protein 1 [Source:MarkerSymbol;Acc:MGI:1309464]	signal transduction/Receptor	1.6	-1.5
ENSMUST00000024979	mitogen-activated protein kinase 8 interacting protein 3	signal transduction/Receptor	1.8	-1.5
ENSMUST00000016672	MAP kinase-activated protein kinase 2 [Source:MarkerSymbol;Acc:MGI:109298]	signal transduction/Receptor	2.1	-1.4
ENSMUST00000019611	RAC/CDC42 exchange factor	signal transduction/Receptor	2.0	-1.9
ENSMUST00000049239	mitogen-activated protein kinase kinase kinase kinase 5 [Source:MarkerSymbol;Acc:MGI:1925503]	signal transduction/Receptor	1.5	-1.3
ENSMUST00000055100	guanine nucleotide binding protein (G protein), gamma 2 subunit	signal transduction/Receptor	3.0	-3.3
ENSMUST00000043624	thyroid hormone receptor associated protein 1	signal transduction/Receptor	1.9	-1.3
ENSMUST00000049149	low density lipoprotein receptor-related protein 1 [Source:MarkerSymbol;Acc:MGI:96828]	signal transduction/Receptor	1.3	-1.5
ENSMUST00000032322	low density lipoprotein receptor-related protein 6 [Source:MarkerSymbol;Acc:MGI:1298218]	signal transduction/Receptor	1.4	-1.6
ENSMUST00000020027	tumor differentially expressed 2	signal transduction/Receptor	2.8	-1.3
ENSMUST00000064921	epidermal growth factor receptor [Source:MarkerSymbol;Acc:MGI:95294]	signal transduction/Receptor	1.4	-1.6
ENSMUST00000033642	doublecortin	signal transduction/Receptor	2.0	-3.1
ENSMUST00000020408	transformed mouse 3T3 cell double minute 2	signal transduction/Receptor	1.3	-1.4
ENSMUST00000027952	plexin A2	signal transduction/Receptor	3.0	-1.7
ENSMUST00000052172	chemokine (C-X-C motif) receptor 4 [Source:MarkerSymbol;Acc:MGI:109563]	signal transduction/Receptor	1.3	-1.5
ENSMUST00000017836	rhomboid, veinlet-like 4 (Drosophila)	signal transduction/Receptor	9.8	-2.7
ENSMUST00000014917	delta-like 1 (Drosophila)	signal transduction/Receptor	2.0	-2.5
ENSMUST00000018313	manic fringe homolog (Drosophila)	signal transduction/Receptor	2.9	-3.2
ENSMUST00000030571	brain-specific angiogenesis inhibitor 2 [Source:MarkerSymbol;Acc:MGI:2451244]	signal transduction/Receptor	1.7	-2.5
ENSMUST00000023015	wingless-related MMTV integration site 7B [Source:MarkerSymbol;Acc:MGI:98962]	signal transduction/Receptor	2.4	-2.5
ENSMUST00000054294	frizzled homolog 1 (Drosophila) [Source:MarkerSymbol;Acc:MGI:1196625]	signal transduction/Receptor	1.7	-2.0
ENSMUST00000040687	Tax1 (human T-cell leukemia virus type I) binding protein 3 [Source:MarkerSymbol;Acc:MGI:1923531]	signal transduction/Receptor	1.4	-1.3
ENSMUST00000072741	frizzled homolog 3 (Drosophila)	signal transduction/Receptor	1.4	-1.5
ENSMUST00000034566	DIX domain containing 1 [Source:MarkerSymbol;Acc:MGI:2679721]	signal transduction/Receptor	2.0	-3.8
ENSMUST00000010038	RIKEN cDNA 1110031B06 gene (1110031B06Rik), mRNA [Source:RefSeq_dna;Acc:NM_144521]	transport	1.3	-1.4
ENSMUST00000023040	peroxisomal integral membrane protein 47 [Source:MarkerSymbol;Acc:MGI:1931461]	transport	1.5	-1.3
ENSMUST00000034830	cellular retinoic acid binding protein I	transport	1.8	-2.1
ENSMUST00000021717	kinesin 2 [Source:MarkerSymbol;Acc:MGI:107978]	transport	3.9	-2.4
ENSMUST00000025110	synaptotagmin IV [Source:MarkerSymbol;Acc:MGI:101759]	transport	1.8	-2.6
ENSMUST00000050000	syntaxin binding protein 1	transport	2.1	-4.6
ENSMUST00000024575	t-complex protein 10c [Source:MarkerSymbol;Acc:MGI:98543]	unknown	2.1	-1.5
ENSMUST00000051937	RAS-like, family 11, member B	unknown	2.0	-1.3
ENSMUST00000021466	spastic paraplegia 3A homolog (human) [Source:MarkerSymbol;Acc:MGI:1921241]	unknown	1.4	-1.9

ENSMUST00000086500	PREDICTED: similar to microtubule-associated protein 1 light chain 3 beta [Source:RefSeq_peptide_predicted;Acc:XP_486190]	unknown	1.5	-1.3
ENSMUST00000040877	prion protein dublet [Source:MarkerSymbol;Acc:MGI:1346999]	unknown	2.2	-1.4
ENSMUST00000077548	CTTNBP2 N-terminal like [Source:MarkerSymbol;Acc:MGI:1933137]	unknown	1.3	-1.7
ENSMUST00000088929	limb expression 1 homolog (chicken) [Source:MarkerSymbol;Acc:MGI:1913893]	unknown	1.3	-1.4
ENSMUST00000054776	pleckstrin homology domain containing, family F (with FYVE domain) member 2	unknown	2.0	-1.6
ENSMUST00000046945	paralemmin [Source:MarkerSymbol;Acc:MGI:1261814]	unknown	1.3	-1.9
ENSMUST00000040967	ATP-binding cassette, sub-family B (MDR/TAP), member 9	unknown	2.0	-1.6
ENSMUST00000087029	rap2 interacting protein x [Source:RefSeq_peptide;Acc:NP_081806]	unknown	1.7	-4.0
ENSMUST00000034285	Fc receptor-like mucin-like 1	unknown	2.0	-5.4
ENSMUST00000068023	immunoglobulin superfamily, member 4C [Source:MarkerSymbol;Acc:MGI:2449088]	unknown	2.7	-2.7
ENSMUST00000045473	transmembrane and coiled-coil domains 2 [Source:MarkerSymbol;Acc:MGI:1916125]	unknown	1.4	-1.7
ENSMUST00000041555	RIKEN cDNA 2610528K11 gene (2610528K11Rik), mRNA [Source:RefSeq_dna;Acc:NM_175184]	unknown	1.4	-1.5
ENSMUST00000054343	AKT1 substrate 1 (proline-rich) [Source:MarkerSymbol;Acc:MGI:1914855]	unknown	2.5	-1.3
ENSMUST00000031391	B-cell CLL/lymphoma 7A	unknown	1.3	-1.8
ENSMUST00000025092	RIKEN cDNA 2810417M05 gene [Source:MarkerSymbol;Acc:MGI:1915277]	unknown	2.0	-2.8
ENSMUST00000048292	cat eye syndrome chromosome region, candidate 2 homolog (human)	unknown	1.7	-1.4
ENSMUST00000093290	pellino 1 [Source:MarkerSymbol;Acc:MGI:1914495]	unknown	2.1	-1.7
ENSMUST00000091518	Gag protein	unknown	2.9	-2.8
ENSMUST00000034145	RIKEN cDNA 4933431N12 gene (4933431N12Rik), mRNA [Source:RefSeq_dna;Acc:NM_027758]	unknown	2.2	-2.1
ENSMUST00000038841	RIKEN cDNA 4933402J24 gene (4933402J24Rik), mRNA [Source:RefSeq_dna;Acc:NM_028940]	unknown	1.7	-1.6
ENSMUST00000031094	TBC1 domain family, member 14 [Source:MarkerSymbol;Acc:MGI:1098708]	unknown	2.4	-2.4
ENSMUST00000061904	2 cells egg cDNA, RIKEN full-length enriched library, clone:B020037P20 product:E1A binding protein p400, full insert sequence. [Source:I	unknown	1.5	-2.3
ENSMUST00000085539	MKIAA1250 protein (Fragment). [Source:Uniprot/SPTREMBL;Acc:Q80TG7]	unknown	1.4	-1.3
ENSMUST00000057935	RIKEN cDNA B430110G05 gene (B430110G05Rik)	unknown	1.5	-1.6
ENSMUST00000058667	leucine rich repeat containing 4B [Source:RefSeq_peptide;Acc:NP_937893]	unknown	2.3	-1.6
ENSMUST00000051145	WD repeat domain 47 [Source:MarkerSymbol;Acc:MGI:2139593]	unknown	1.8	-1.5
ENSMUST00000050001	RIKEN full-length enriched libraryA230015N21	unknown	3.2	-1.7
ENSMUST00000037795	RIKEN cDNA 6330407J23 gene (6330407J23Rik), mRNA [Source:RefSeq_dna;Acc:NM_026138]	unknown	1.5	-1.9
ENSMUST00000043618	hect (homologous to the E6-AP (UBE3A) carboxyl terminus) domain and RCC1 (CHC1)-like domain (RLD) 1 [Source:MarkerSymbol;Acc:I	unknown	1.9	-1.3
ENSMUST00000086034	CAR-like membrane protein	unknown	1.4	-1.3
ENSMUST00000023219	F-box and leucine-rich repeat protein 6	unknown	1.6	-1.3
ENSMUST00000033519	t-complex-associated-testis-expressed 1-like [Source:MarkerSymbol;Acc:MGI:1914367]	unknown	1.8	-1.4
ENSMUST00000067880	a disintegrin and metallopeptidase domain 10 [Source:MarkerSymbol;Acc:MGI:109548]	unknown	1.7	-1.3
ENSMUST00000044297	insulin-like growth factor binding protein-like 1 [Source:MarkerSymbol;Acc:MGI:1933198]	unknown	1.6	-3.7
ENSMUST00000025663	transmembrane protein 2	unknown	2.2	-1.3
ENSMUST00000056756	opposite strand transcription unit to Stag3	unknown	1.8	-1.5
ENSMUST00000021177	SEC14-like 1 (S. cerevisiae)	unknown	2.9	-1.4
ENSMUST00000035121	angiomotin like 2 [Source:MarkerSymbol;Acc:MGI:1929286]	unknown	1.4	-1.3
ENSMUST00000023251	lymphocyte antigen 6 complex, locus E [Source:MarkerSymbol;Acc:MGI:106651]	unknown	2.1	-1.8
ENSMUST00000089123	RIKEN cDNA 5430432M24 gene (5430432M24Rik)	unknown	9.1	-3.3
ENSMUST00000085261	RIKEN cDNA 6330442E10 gene (6330442E10Rik), mRNA [Source:RefSeq_dna;Acc:NM_178745]	unknown	1.3	-2.1
ENSMUST00000064444	gene model 50, (NCBI) [Source:MarkerSymbol;Acc:MGI:2684896]	unknown	1.4	-1.5
ENSMUST00000093088	genetic suppressor element 1	unknown	1.7	-1.5
ENSMUST00000027837	loop tail associated protein [Source:MarkerSymbol;Acc:MGI:2135272]	unknown	1.4	-1.4
ENSMUST00000061262	podocalyxin-like 2 [Source:MarkerSymbol;Acc:MGI:2442488]	unknown	2.0	-2.9
ENSMUST00000093335	Integrase (Fragment). [Source:Uniprot/SPTREMBL;Acc:Q811N0]	unknown	1.6	-2.1
ENSMUST00000045270	core-binding factor, runt domain, alpha subunit 2, translocated to, 2 homolog (human)	unknown	2.6	-1.8
ENSMUST00000043356	RIKEN cDNA 0610010F05	unknown	1.9	-2.0
ENSMUST00000039506	immunoglobulin superfamily, member 8 [Source:MarkerSymbol;Acc:MGI:2154090]	unknown	1.7	-2.6

Mash1 targets

Ensembl Transcript ID	Description	Gene Ontology	Mash1GOF	Mash1-/-
ENSMUST00000034472	junction adhesion molecule 3	cell adhesion	1.3	-1.5
ENSMUST00000035129	Eph receptor B1	cell adhesion	1.5	-1.5
ENSMUST00000035499	neighbor of Punc E11	cell adhesion	1.6	-2.1
ENSMUST00000020692	B-cell translocation gene 2, anti-proliferative	cell cycle	1.5	-1.7
ENSMUST00000021903	growth arrest and DNA-damage-inducible 45 gamma	cell cycle	1.4	-5.6
ENSMUST00000022639	neurofilament, light polypeptide	cytoskeleton	2.3	-1.8
ENSMUST00000027687	ubiquitin-conjugating enzyme E2T (putative)	enzyme	1.7	-1.5
ENSMUST00000045043	RAN binding protein 9	protein binding	1.3	-1.3
ENSMUST00000050706	glucocorticoid induced transcript 1	protein binding	2.1	-1.9
ENSMUST00000020243	achaete-scute complex homolog-like 1	regulation of transcription	39.2	-39.5
ENSMUST00000025081	zinc finger homeobox 1a	regulation of transcription	1.7	-1.4
ENSMUST00000029852	LIM homeobox protein 8	regulation of transcription	1.5	-2.0
ENSMUST00000036060	ISL1 transcription factor, LIM/homeodomain	regulation of transcription	2.4	-1.8
ENSMUST00000049621	hairy and enhancer of split 5	regulation of transcription	1.5	-10.5

ENSMUST00000053491	POU domain, class 3, transcription factor 1	regulation of transcription	1.6	-1.4
ENSMUST00000086851	hairy and enhancer of split 6	regulation of transcription	2.4	-2.3
ENSMUST00000001386	ELAV like 4 (Hu antigen D)	RNA binding	1.7	-1.7
ENSMUST00000025363	heparin-binding EGF-like growth factor	signal transduction/Receptor	1.4	-1.4
ENSMUST00000027748	regulator of G-protein signaling 16	signal transduction/Receptor	4.6	-1.4
ENSMUST00000033133	regulator of G-protein signalling 10	signal transduction/Receptor	2.1	-1.8
ENSMUST00000041776	Regulator of G-protein signaling 8	signal transduction/Receptor	7.4	-1.7
ENSMUST00000065587	chemokine orphan receptor 1	signal transduction/Receptor	1.4	-1.4
ENSMUST00000073392	retinitis pigmentosa GTPase regulator	signal transduction/Receptor	1.5	-1.3
ENSMUST00000090679	tachykinin 1	signal transduction/Receptor	2.6	-1.4
ENSMUST00000018478	kinase suppressor of ras	signal transduction/Receptor	1.8	-1.4
ENSMUST00000018313	manic fringe homolog	signal transduction/Receptor	2.8	-2.8
ENSMUST00000017836	rhomboid, veinlet-like 4	signal transduction/Receptor	8.2	-2.7
ENSMUST00000031555	lunatic fringe gene homolog	signal transduction/Receptor	1.8	-4.8
ENSMUST00000014917	delta-like 1	signal transduction/Receptor	2.0	-3.6
ENSMUST00000059285	Notch-regulated ankyrin repeat protein	signal transduction/Receptor	1.9	-3.2
ENSMUST00000082054	regulating synaptic membrane exocytosis 2	transport	1.3	-1.3
ENSMUST00000025178	vacuolar protein sorting 52 (yeast)	transport	1.8	-1.5
ENSMUST00000030317	podoplanin	transport	2.2	-4.1
ENSMUST00000042329	nuclear transport factor 2-like export factor 2	transport	1.3	-1.5
ENSMUST00000045738	Viaat, solute carrier family 32 member 1	transport	1.7	-1.3
ENSMUST00000044048	heat shock protein, alpha-crystallin-related, B6	unknown	1.3	-1.3
ENSMUST00000025058	ankyrin repeat and SAM domain containing 1	unknown	4.2	-1.8
ENSMUST00000028257	grancalcin	unknown	2.5	-1.8
ENSMUST00000039506	immunoglobulin superfamily, member 8	unknown	1.6	-1.7
ENSMUST00000042499	tripartite motif-containing 59	unknown	1.3	-1.4
ENSMUST00000058009	RIKEN cDNA 6330500D04	unknown	1.3	-1.5
ENSMUST00000065118	PREDICTED: hypothetical protein LOC76980	unknown	1.9	-1.4
ENSMUST00000069180	RIKEN cDNA 2310047A01	unknown	1.6	-2.2
ENSMUST00000085669	RIKEN cDNA 5730507C01	unknown	1.3	-1.4
ENSMUST00000087702	RIKEN cDNA 2210010L05 gene	unknown	1.4	-1.3

Common targets

**Ensembl Transcript ID**	**Description**	**Gene Ontology**	**Ngn2GOF**	**Mash1GOF**	**Mash1-/-**	**Mash1-/-;Ngn2-/-**
ENSMUST00000035499	neighbor of Punc E11	cell adhesion	1.4	1.6	-2.1	-1.6
ENSMUST00000020692	B-cell translocation gene 2, anti-proliferative	cell cycle	2.9	1.5	-1.7	-1.4
ENSMUST00000049621	hairy and enhancer of split 5	regulation of transcription	1.5	1.5	-10.5	-20.8
ENSMUST00000086851	hairy and enhancer of split 6	regulation of transcription	6.0	2.4	-2.3	-2.9
ENSMUST00000001386	ELAV like 4 (Hu antigen D)	RNA binding	1.9	1.7	-1.7	-2.7
ENSMUST00000065587	chemokine orphan receptor 1	signal transduction/Receptor	1.8	1.4	-1.4	-5.1
ENSMUST00000017836	rhomboid, veinlet-like 4	signal transduction/Receptor	9.8	8.2	-2.7	-2.7
ENSMUST00000018313	manic fringe homolog	signal transduction/Receptor	2.9	2.8	-2.8	-3.2
ENSMUST00000014917	delta-like 1	signal transduction/Receptor	2.0	2.0	-3.6	-2.5
ENSMUST00000039506	immunoglobulin superfamily, member 8	unknown	1.7	1.6	-1.7	-2.6
