# Supplementary material for: Characterization of the proneural gene regulatory network during mouse telencephalon development
Source: BMC Biol. 2008 Mar 31;6:15. doi: 10.1186/1741-7007-6-15 (PMC2330019; doi:10.1186/1741-7007-6-15)
Supplement: Additional file 4 — List of targets analyzed using in situ hybridization in wild-type and mutant embryos ( Ngn2-/- and Mash1-/-), and of embryos overexpressing Ngn2 or Mash1. [file 1741-7007-6-15-S4.pdf]

**Ensembl Transcript ID**	**Description**	**Ngn2GOF**	**Mash1-/-;Ngn2-/-**	***In situ* Ngn2-/- Cx**	***In situ* Ngn2GOF**
ENSMUST00000059794	Nscl1	1.7	-18.0	decrease	increase
ENSMUST00000077225	Zfp238	1.3	-1.9	decrease	increase
ENSMUST00000001386	HuD	1.9	-2.7	decrease	increase
ENSMUST00000020027	Serinc1	2.8	-1.3	no expression	na
ENSMUST00000017836	Ventrhoid	9.8	-2.7	decrease	increase
ENSMUST00000018313	Mfng	2.9	-3.2	decrease	increase
ENSMUST00000023015	Wnt7B	2.4	-2.5	no change	na
ENSMUST00000054776	Plekhf2	2.0	-1.6	no expression	na

**Ensembl Transcript ID**	**Description**	**Mash1GOF**	**Mash1-/-**	***In situ* Mash1-/- Bg**	***In situ* Mash1 GOF**
ENSMUST00000021903	Gadd45g	1.4	-5.6	decrease	increase
ENSMUST00000029852	Lhx8	1.5	-2.0	upregulated in VZ	na
ENSMUST00000001386	HuD	1.7	-1.7	decrease	increase
ENSMUST00000027748	Rgs16	4.6	-1.4	no change	na
ENSMUST00000018313	Mfng	2.8	-2.8	decrease	increase
ENSMUST00000017836	Ventrhoid	8.2	-2.7	decrease	increase
ENSMUST00000059285	Nrarp	1.9	-3.2	decrease	no change
ENSMUST00000030317	Gp38/Podoplanin	2.2	-4.1	decrease	increase
