# Supplementary material for: Characterization of the proneural gene regulatory network during mouse telencephalon development
Source: BMC Biol. 2008 Mar 31;6:15. doi: 10.1186/1741-7007-6-15 (PMC2330019; doi:10.1186/1741-7007-6-15)
Supplement: Additional file 5 — Quantitative PCR analysis of predicted Mash1 targets in P19 cells following transfection of a Mash1 expression vector. [file 1741-7007-6-15-S5.pdf]

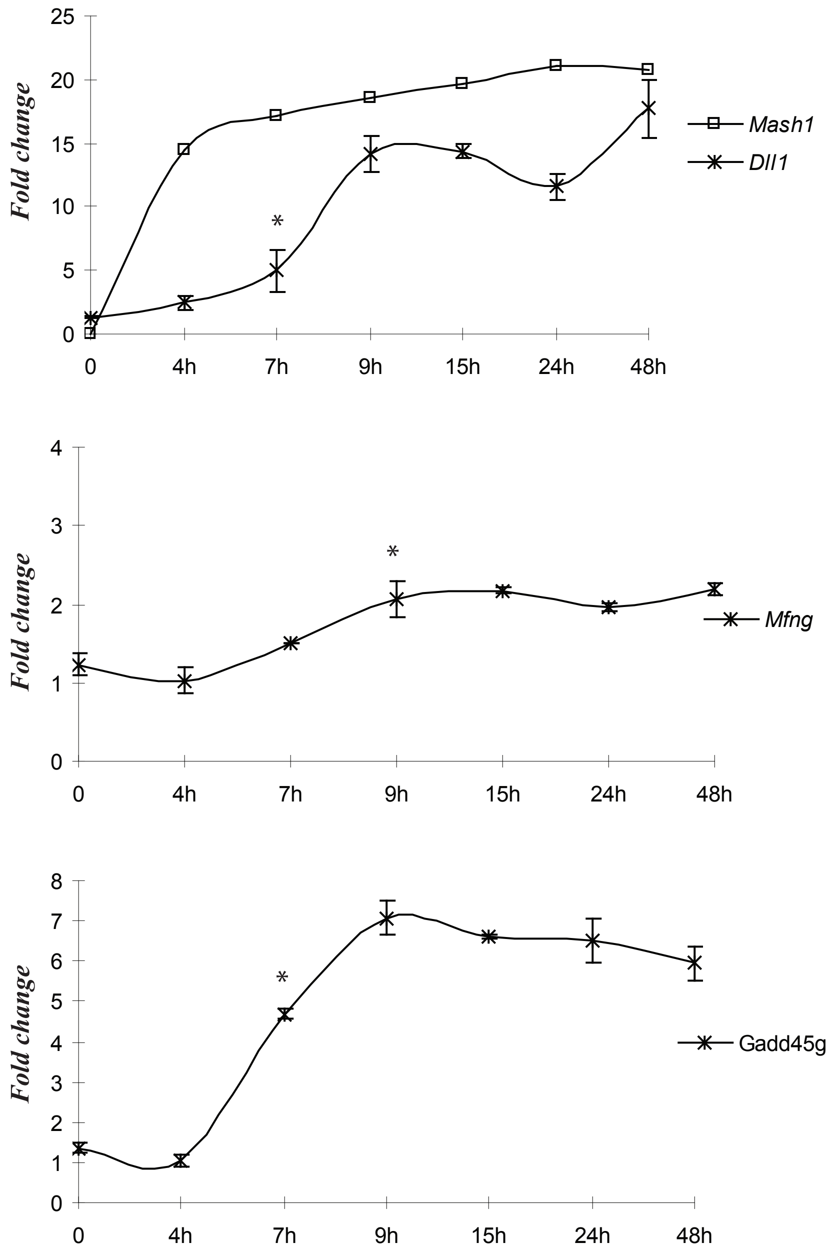

Additional file 5. Quantitative PCR analysis of predicted Mash1 targets in P19 cells following transfection of a Mash1 expression vector. A significant upregulation of Dll1, Mfng and Gadd45g is detected between 7hrs and 9hrs after transfection (*). Fold change were normalized with HMBS (hydroxymethylbilane synthase) as a reference.
