# Supplementary material for: Characterization of the proneural gene regulatory network during mouse telencephalon development
Source: BMC Biol. 2008 Mar 31;6:15. doi: 10.1186/1741-7007-6-15 (PMC2330019; doi:10.1186/1741-7007-6-15)
Supplement: Additional file 6 — Quantification of literature-based network structure. [file 1741-7007-6-15-S6.doc]

Quantification of literature-based network structure

| **Linkage** | **β (mean value)*** | **Percent<0** |
| --- | --- | --- |
| Mash1 to Dlx1 | 0.22 | 2.1 |
| Mash1 to Dlx2 | 0.46 | 2.2 |
| Dlx1 to Dlx5 | 0.82 | 0 |
| Dlx2 to Dlx5 | 0.23 | 0 |
| Neurog2 to Eomes | 0.42 | 0.2 |
| Dlx5 to Gad1 | 1.10 | 0 |
| Dlx5 to Gad2 | 0.13 | 2.1 |
| Dll1 to Hes1 | 0.37 | 0 |
| Dll1 to Hes5 | 0.98 | 0 |
| Neurog1 to Neurod2 | 0.69 | 0 |
| Neurog2 to Neurod2 | 0.57 | 3.5 |
| Neurog2 to Neurod6 | 0.83 | 0 |
| Neurod1 to Otx1 | 0.08 | 33.6 |
| Neurod1 to Robo1 | 0.32 | 0.2 |
| Pax6 to Satb2 | -0.56 | 99 |
| Eomes to Tbr1 | 1.13 | 0 |
| Neurog1 to Neurod1 | 0.44 | 0.3 |
| Neurog2 to Neurod1 | 0.44 | 0 |
| Pax6 to Neurog1 | -0.27 | 97.6 |
| Neurog2 to Neurog1 | 0.12 | 11.6 |
| Pax6 to Neurog2 | 1.52 | 0 |
| Wnt7b to Neurog2 | 0.39 | 1.8 |
| Neurog2 to Mash1 | 1.19 | 4.2 |
| Neurod1 to Etv1 | -0.66 | 96.4 |
| Neurod1 to Nscl | 1.24 | 0 |
| Neurog1 to Nscl | 0.90 | 0 |
| Neurog2 to Nscl | -0.32 | 97.1 |
| Neurod1 to Id2 | 0.74 | 0 |
| Pax6 to Pou6f1 | -0.26 | 89.9 |
| Neurod1 to Slc17a6 | 0.98 | 0 |
| Dlx5 to Viaat | 0.32 | 0.1 |
| Dlx5 to Slc6a1 | 0.31 | 0 |
| Dlx1 to Arx | 0.38 | 0 |
| Dlx2 to Arx | -0.01 | 54.8 |
| Neurog2 to Dll1 | -0.15 | 70 |
| Mash1 to Dll1 | 0.16 | 4.5 |

*****Quantification was based on a linear relationship between genes that are linked (See Methods). The mean value for β is indicated along with the percentage of Marcov Chain Monte Carlo (MCMC) simulations that gave a value for β less than or equal to zero, indicating the significance of the linkage based on the microarray datasets. If β *_ji_* is 0, the model based on the microarray gene expression data estimates there is no relationship between parent and child gene expression. If β *_ji_*>0, then for every 1 unit change in the parent gene, the child gene will change by a factor of 2 β *_ji_* times its baseline value. Therefore, β *_ji_*=2 would imply a 4 fold change for a doubling of the parent gene. If the linkage is an inhibition, then for every 1 unit change in the parent gene, the child gene will decrease by a factor of 2 times the baseline. For example, β *_ji_*=2 implies a 4 fold decrease (1/4) for a doubling of the parent. The percentage of MCMC simulations that estimate β *_ji_* below or at 0 is used to determine the significance of each connection (< 5%). 500,000 MCMC iterations were performed.
