# Supplementary material for: Characterization of the proneural gene regulatory network during mouse telencephalon development
Source: BMC Biol. 2008 Mar 31;6:15. doi: 10.1186/1741-7007-6-15 (PMC2330019; doi:10.1186/1741-7007-6-15)
Supplement: Additional file 7 — Determination of Ngn2 and Mash1 consensus binding sites. [file 1741-7007-6-15-S7.pdf]

Ngn2 Consensus

NeuroD E1	CCATATGG	Ref1
NeuroD E2	ACAGATGG	Ref1
NeuroD2	ACAGATGG	Ref2
Dll1 E1	GCAGATGG	Ref3
Dll1 E2	CCAAATGG	Ref3
Consensus	NCANATGG	

Mash1 Consensus

Brd E1	GCAGGTGT	Ref4
Sca E1	GCAGGTGG	Ref4
E(spl)m7 E2	GCAGCTGT	Ref4
Consensus 1	GCAGSTGK	Ref4,5
Dll1 E3	CAGGTG	Ref3
Dll1 E4	CAGCTG	Ref3
Consensus 2	CAGSTG	Ref3,6

Ref1: Huang HP, Liu M, El-Hodiri HM, Chu K, Jamrich M, Tsai MJ. Mol Cell Biol. 2000 May;20(9):3292-307

Ref2: Lin CH, Stoeck J, Ravanpay AC, Guillemot F, Tapscott SJ, Olson JM. Dev Biol. 2004 Jan 1;265(1):234-45

Ref3: Castro, D.S., et al. Dev Cell, 2006. 11(6): p. 831-844

Ref4: Singson, A., M. W. Leviten, A. G. Bang, X. H. Hua, and J. W. Posakony. Genes Dev. 8:2058-2071

Ref5: Powell LM, Zur Lage PI, Prentice DR, Senthinathan B, Jarman AP. Mol Cell Biol. 2004 Nov;24(21):9517-26

Ref6: Hu Y, Wang T, Stormo GD, Gordon JI. Proc Natl Acad Sci U S A. 2004 Apr 13;101(15):5559-64
