# Supplementary material for: Characterization of the proneural gene regulatory network during mouse telencephalon development
Source: BMC Biol. 2008 Mar 31;6:15. doi: 10.1186/1741-7007-6-15 (PMC2330019; doi:10.1186/1741-7007-6-15)
Supplement: Additional file 8 — List of Ngn2, Mash1, and common target genes examined in co-factor/co-regulator analyses. [file 1741-7007-6-15-S8.pdf]

Ngn2 target
Atp9a
Crabp1
Dusp14
Acpl2
Corob2
Fzd1
Auts2
Nhlh1
Bai2
Wnt7b
Bhlhb5

Mash1 target
Rarb
Pou3f1
Dlx1
Stk33
Pdpn
Insm1
Lmo1
Isl1
Anks1
Lfng
Gadd45g
Glcci1
Lhx8
Gca

Mash1/Ngn2 target
Mfng
Hes6
Dll1
Hes5
Btg2
Elavl4
