# Supplementary material for: Characterization of the proneural gene regulatory network during mouse telencephalon development
Source: BMC Biol. 2008 Mar 31;6:15. doi: 10.1186/1741-7007-6-15 (PMC2330019; doi:10.1186/1741-7007-6-15)
Supplement: Additional file 10 — Corroboration of predicted co-factors, co-regulators and related proteins via interrogation of online databases. [file 1741-7007-6-15-S10.doc]

**Corroboration of predicted co-factors, co-regulators and related proteins via interrogation of online databases**

| **Predicted**  **Co-factor/Co-regulator** | ***In situ* hybridization (genepaint.org and stjudebgem.org)** |
| --- | --- |
| Sox9 | Strong expression in the ventricular zone of the dorsal and ventral telencephalon (E14.5, genepaint.org) |
| Sox8 | Strong expression in the ventricular zone of the dorsal and ventral telencephalon (E14.5, genepaint.org) |
| Sox10 | Very weak expression in the ventricular zone of the ventral telencephalon (E15.5, stjudebgem.org) |
| Crebbp | Weak expression in the ventricular zone of the dorsal and ventral telencephalon (E14.5, genepaint.org) |
| Creb1 | Strong expression throughout the dorsal and ventral telencephalon (E14.5, genepaint.org) |
| Tcf4 | Moderate expression in the ventricular zone of the dorsal and ventral telencephalon, strong in cortical mantle zone (E14.5, genepaint.org) |
| Lef1 | Weak expression in the ventricular zone of the dorsal telencephalon (E14.5, genepaint.org) |
| Mef2a | No detectable expression in the telencephalon (E14.5, genepaint.org) |
| Mef2b | Weak expression in the ventricular zone of the dorsal telencephalon (E14.5, genepaint.org) |
| Mef2c | Strong expression in the mantle zone of the dorsal and ventral telencephalon (E14.5, genepaint.org) |
| Yy1 | - |
| Pou6f1 | Strong expression throughout the dorsal and ventral telencephalon (E14.5, genepaint.org) |
| Pou2f1 | Moderate expression in the ventricular zone and weak expression in the rest of the dorsal and ventral telencephalon (E14.5, genepaint.org) |
| Pou3f1 | Strong expression throughout the dorsal telencephalon and weaker expression throughout the ventral telencephalon (E15.5, stjudebgem.org) |
| Pou3f4 | Moderate expression in the subventricular zone of the ventral telencephalon (E14.5, genepaint.org) |
| Nfya | - |
| Nfyb | Moderate expression in the mantle zone of the dorsal telencephalon (E14.5, genepaint.org) |
| Tef | Weak expression in the ventricular zone of the dorsal and ventral telencephalon (E14.5, genepaint.org) |
| Hes1 | Strong expression in the ventricular zone of the dorsal and ventral telencephalon (E15.5, stjudebgem.org) |
| Egr1 | No detectable expression in the telencephalon (E14.5, genepaint.org) |
| E2f1 | Strong expression in the ventricular zone of the dorsal and ventral telencephalon (E14.5, genepaint.org) |
