# Supplementary figures and images for: Characterization of the proneural gene regulatory network during mouse telencephalon development
Source: BMC Biol. 2008 Mar 31;6:15. doi: 10.1186/1741-7007-6-15 (PMC2330019; doi:10.1186/1741-7007-6-15)

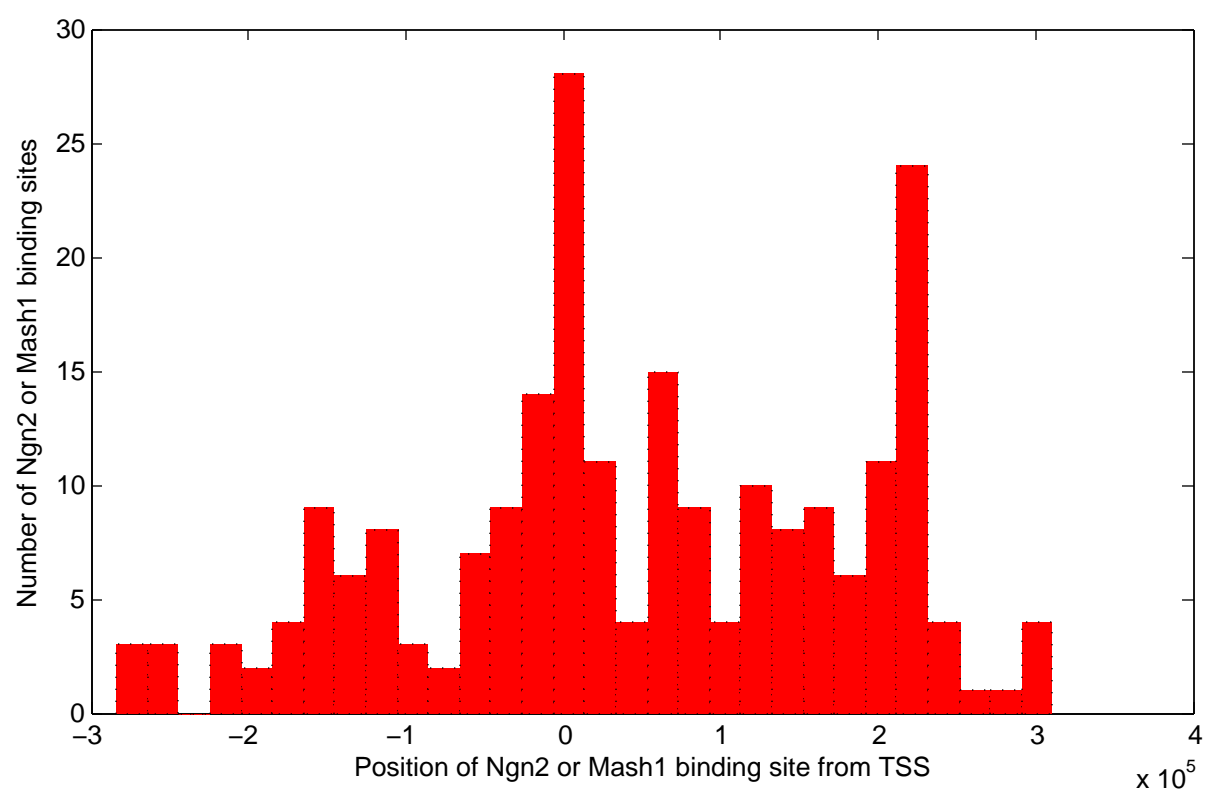

Supplement: Additional file 11 — Matrix of connectivity of algorithm-based gene regulatory network structure for dorsal and ventral telencephalon development. [file 1741-7007-6-15-S11.pdf]
