# Supplementary material for: Characterization of the proneural gene regulatory network during mouse telencephalon development
Source: BMC Biol. 2008 Mar 31;6:15. doi: 10.1186/1741-7007-6-15 (PMC2330019; doi:10.1186/1741-7007-6-15)
Supplement: Supplementary file 11 [file 1741-7007-6-15-S9.doc]

CONFAC analysis using Mash1 and Common targets

Control data: Random250-cons.confac

Cut-offs:

Mean Difference: 0.50
P-value: 0.05

Factor	Mean - Sample	Mean - Control	Mean Difference	P-value	Std-Dev - Sample	Std-Err - Sample	Std-Dev - Control	Std-Err - Control
TCF4	7.345	0.521	6.824	0.000	7.650	1.421	1.007	0.092
CREBP1	1.000	0.244	0.756	0.002	2.053	0.381	0.759	0.070
HES1	19.931	1.664	18.267	0.000	18.274	3.393	2.297	0.211
OCT	2.483	0.076	2.407	0.000	4.315	0.801	0.266	0.024
TAXCREB	0.586	0.084	0.502	0.000	0.780	0.145	0.308	0.028
SOX	2.793	0.193	2.600	0.000	2.896	0.538	0.541	0.050
NFY	17.379	2.017	15.363	0.000	16.826	3.124	2.928	0.268
E2F	17.241	3.387	13.855	0.000	16.416	3.048	5.809	0.533
TCF11MAFG	0.793	0.034	0.759	0.000	0.940	0.175	0.181	0.017
AMEF2	2.828	0.109	2.718	0.000	4.027	0.748	0.339	0.031
POU3F2	1.069	0.076	0.993	0.000	2.069	0.384	0.296	0.027
YY1	2.621	0.252	2.369	0.000	2.484	0.461	0.540	0.050
MEF2	6.517	0.412	6.105	0.000	8.822	1.638	1.278	0.117
E2F1DP1	4.103	0.697	3.406	0.000	4.562	0.847	1.078	0.099
E2F1DP1RB	1.552	0.269	1.283	0.000	1.863	0.346	0.673	0.062
E2F1DP2	1.828	0.286	1.542	0.000	2.285	0.424	0.626	0.057
E2F1	22.000	4.336	17.664	0.000	23.661	4.394	6.703	0.615
TEF	2.655	0.076	2.580	0.000	4.345	0.807	0.348	0.032
**EBOX sites**								
TAL1BETAITF2	1.310	0.050	1.260	0.000	2.002	0.372	0.255	0.023
HAND1E47	26.621	2.000	24.621	0.000	24.311	4.514	2.487	0.228
HEN1	2.724	0.193	2.531	0.000	3.401	0.631	0.773	0.071
AREB6	7.966	0.790	7.176	0.000	7.094	1.317	1.484	0.136
HEB	5.069	0.756	4.313	0.000	5.113	0.949	1.025	0.094
TAL1BETAE47	4.310	0.261	4.050	0.000	3.828	0.711	0.574	0.053
TAL1	4.310	0.462	3.848	0.000	3.626	0.673	0.790	0.072
E12	6.241	0.454	5.788	0.000	5.145	0.955	0.810	0.074
MYOD	10.448	0.706	9.742	0.000	8.749	1.625	1.265	0.116
TAL1ALPHAE47	4.276	0.244	4.032	0.000	3.807	0.707	0.552	0.051
AP4	2.483	0.185	2.298	0.000	2.600	0.483	0.431	0.040

CONFAC analysis using Ngn2 targets

Control data: Random250-cons.confac

Cut-offs:

Mean Difference: 0.50
P-value: 0.1

Factor	Mean - Sample	Mean - Control	Mean Difference	P-value	Std-Dev - Sample	Std-Err - Sample	Std-Dev - Control	Std-Err - Control
HES1	67.125	1.664	65.461	0	59.77	21.132	2.297	0.211
MMEF2	87.875	0.521	87.354	0	101.685	35.951	1.111	0.102
MEF2	18.5	0.412	18.088	0	21.541	7.616	1.278	0.117
LEF1	23.25	0.504	22.746	0	22.632	8.002	0.938	0.086
SOX9	32.5	0.58	31.92	0	36.198	12.798	1.004	0.092
E2F1DP2	1.875	0.286	1.589	0.001	2.031	0.718	0.626	0.057
E2F1DP1	3.875	0.697	3.178	0.003	4.051	1.432	1.078	0.099
E2F	12.25	3.387	8.863	0.005	10.82	3.825	5.809	0.533
E2F1	11.625	4.336	7.289	0.02	11.25	3.977	6.703	0.615
POU6F1	4	0.05	3.95	0	4.66	1.648	0.315	0.029
CREBATF	27.25	0.664	26.586	0	24.61	8.701	1.13	0.104
OCT	5.125	0.076	5.049	0	5.436	1.922	0.266	0.024
CREB	56.5	2.017	54.483	0	52.623	18.605	3.596	0.33
POU1F1	72	0.378	71.622	0	77.062	27.246	0.844	0.077
AMEF2	9.25	0.109	9.141	0	10.66	3.769	0.339	0.031
POU3F2	1.5	0.076	1.424	0	2.449	0.866	0.296	0.027
CEBP	47.875	0.437	47.438	0	48.496	17.146	0.777	0.071
LEF1TCF1	24.25	0.563	23.687	0	24.72	8.74	1.162	0.107
TCF4	32.375	0.521	31.854	0	34.55	12.215	1.007	0.092
NFY	53	2.017	50.983	0	49.811	17.611	2.928	0.268
TCF1P	67.625	1.134	66.491	0	68.145	24.093	1.631	0.15
TEF	7.25	0.076	7.174	0	8.972	3.172	0.348	0.032
TCF11MAFG	1.375	0.034	1.341	0	1.768	0.625	0.181	0.017
CEBPGAMMA	5	0.101	4.899	0	5.928	2.096	0.377	0.035
YY1	8.25	0.252	7.998	0	6.84	2.418	0.54	0.05
Oct1	282.875	1.79	281.085	0	294.997	104.297	3.38	0.31
**EBOX sites**								
HEB	4.125	0.756	3.369	0	2.8	0.99	1.025	0.094
HAND1E47	132.875	2	130.875	0	103.968	36.758	2.487	0.228
AP4	1.75	0.185	1.565	0.01	3.105	1.098	0.431	0.04
HEN1	1.25	0.193	1.057	0	1.282	0.453	0.773	0.071
TAL1	8.625	0.462	8.163	0	6.301	2.228	0.79	0.072
AREB6	12.125	0.79	11.335	0	10.274	3.632	1.484	0.136
E47	19.5	0.605	18.895	0	17.296	6.115	1.277	0.117
LMO2COM	17.375	0.21	17.165	0	18.654	6.595	0.662	0.061
MYOD	20.625	0.706	19.919	0	16.318	5.769	1.265	0.116
TAL1BETAITF2	1.25	0.05	1.2	0	1.753	0.62	0.255	0.023
TAL1ALPHAE47	8.375	0.244	8.131	0	8.434	2.982	0.552	0.051
TAL1BETAE47	8.125	0.261	7.864	0	7.549	2.669	0.574	0.053
E12	17.25	0.454	16.796	0	16.351	5.781	0.81	0.074

CONFAC analysis using Ngn2 target genes versus Mash1 target genes

Cut-offs:

Mean Difference: 1
P-value: 0.05

Factor	Mean - Sample	Mean - Control	Mean Difference	P-value	Std-Dev - Sample	Std-Err - Sample	Std-Dev - Control	Std-Err - Control
TCF1P	25.360	14.586	10.774	0.002	19.962	3.992	17.177	3.190
POU1F1	18.920	9.690	9.230	0.003	14.683	2.937	12.669	2.353
OCT1	75.440	42.345	33.095	0.006	65.143	13.029	51.047	9.479
OCT	3.640	2.483	1.157	0.006	3.187	0.637	4.315	0.801
CEBP	12.800	6.793	6.007	0.006	13.733	2.747	7.903	1.468
LEF1	12.000	6.517	5.483	0.007	10.824	2.165	6.685	1.241
SOX9	13.280	8.103	5.177	0.010	10.683	2.137	8.351	1.551
TCF4	13.800	7.345	6.455	0.010	12.433	2.487	7.650	1.421
MMEF2	28.200	15.000	13.200	0.011	27.515	5.503	21.665	4.023
YY1	5.080	2.621	2.459	0.017	4.142	0.828	2.484	0.461
LEF1TCF1	10.120	6.690	3.430	0.024	7.440	1.488	5.058	0.939
MEF2	11.920	6.517	5.403	0.026	12.861	2.572	8.822	1.638
CREBATF	10.760	7.276	3.484	0.039	8.207	1.641	6.284	1.167
AMEF2	5.040	2.828	2.212	0.039	5.342	1.068	4.027	0.748
TEF	4.160	2.655	1.505	0.041	4.110	0.822	4.345	0.807

Table S2b: Control gene expression of predicted co-regulators (median; GCRMA normalized; log2 scale)					
		MOE		U74	
Ensembl Gene ID	Description	Dorsal	Ventral	Dorsal	Ventral
ENSMUSG00000022521	CREB binding protein [Source:MarkerSymbol;Acc:MGI:1098280]	8.59	5.27	7.52	7.45
ENSMUSG00000027490	E2F transcription factor 1 [Source:MarkerSymbol;Acc:MGI:101941]	5.26	5.99	6.28	6.03
ENSMUSG00000026565	POU domain, class 2, transcription factor 1 [Source:MarkerSymbol;Acc:MGI:101898]	5.95	6.34	7.10	7.30
ENSMUSG00000009739	POU domain, class 6, transcription factor 1 [Source:MarkerSymbol;Acc:MGI:102935]	4.06	4.66	4.65	5.09
ENSMUSG00000000567	SRY-box containing gene 9 [Source:MarkerSymbol;Acc:MGI:98371]	8.00	8.11	6.59	5.88
ENSMUSG00000038418	early growth response 1 [Source:MarkerSymbol;Acc:MGI:95295]	6.61	6.10	1.81	1.92
ENSMUSG00000025958	cAMP responsive element binding protein 1 [Source:MarkerSymbol;Acc:MGI:88494]	7.09	5.45	3.34	3.52
ENSMUSG00000053477	transcription factor 4 [Source:MarkerSymbol;Acc:MGI:98506]	9.18	8.15	10.83	9.44
ENSMUSG00000021264	YY1 transcription factor [Source:MarkerSymbol;Acc:MGI:99150]	5.11	6.15	2.43	2.48
ENSMUSG00000022389	thyrotroph embryonic factor [Source:MarkerSymbol;Acc:MGI:98663]	4.29	4.37	6.16	6.20
ENSMUSG00000020248	nuclear transcription factor-Y beta [Source:MarkerSymbol;Acc:MGI:97317]	7.30	7.85	9.36	8.78
ENSMUSG00000023994	nuclear transcription factor-Y alpha [Source:MarkerSymbol;Acc:MGI:97316]	7.17	6.35	8.54	8.81
ENSMUSG00000030557	myocyte enhancer factor 2A [Source:MarkerSymbol;Acc:MGI:99532]	4.78	5.02	4.75	5.83
ENSMUSG00000027985	lymphoid enhancer binding factor 1 [Source:MarkerSymbol;Acc:MGI:96770]	6.14	4.57	5.43	4.92
ENSMUSG00000053477	transcription factor 4 [Source:MarkerSymbol;Acc:MGI:98506]	9.18	8.15	10.83	9.44
ENSMUSG00000022528	hairy and enhancer of split 1 (Drosophila) [Source:MarkerSymbol;Acc:MGI:104853]	6.84	8.28	5.12	3.23
